# Cryo-EM structure of the SARS-CoV-2 3a ion channel in lipid nanodiscs

**DOI:** 10.1101/2020.06.17.156554

**Published:** 2020-06-25

**Authors:** David M. Kern, Ben Sorum, Christopher M. Hoel, Savitha Sridharan, Jonathan P. Remis, Daniel B. Toso, Stephen G. Brohawn

**Affiliations:** 1 Department of Molecular and Cell Biology, University of California Berkeley, Berkeley, California, 94720, USA; 2 Helen Wills Neuroscience Institute, University of California Berkeley, Berkeley, California, 94720, USA; 3 California Institute for Quantitative Biology (QB3), University of California Berkeley, Berkeley, CA 94720, USA; 4 Molecular Biophysics and Integrative Bioimaging Division, Lawrence Berkeley National Laboratory, Berkeley, CA, 94720, USA

## Abstract

SARS-CoV-2 encodes three putative ion channels: E, 8a, and 3a. In related SARS-CoV-1, 3a is implicated in viral release, inflammasome activation, and cell death and its deletion reduces viral titer and morbidity in animal models, suggesting 3a-targeted therapeutics could treat SARS and COVID-19. However, the structural basis for the function of 3a is unknown. Here, we show that SARS-CoV-2 3a forms large conductance cation channels and present cryo-EM structures of dimeric and tetrameric 3a in lipid nanodiscs. 3a adopts a novel fold and is captured in a closed or inactivated state. A narrow bifurcated exterior pore precludes conduction and leads to a large polar cavity open to the cytosol. 3a function is conserved in a common variant among circulating SARS-CoV-2 that alters the channel pore. We identify 3a-like proteins in all *Alpha-* and *Beta-coronaviruses* that infect bats and humans, suggesting therapeutics targeting 3a could treat a range of coronaviral diseases.

## Introduction

Coronavirus disease 2019 (COVID-19), caused by the SARS-CoV-2 virus, is an ongoing global pandemic. Neutralizing the virus is the focus of a multi-pronged approach, including behavioral, medical, and basic research efforts around the world. Vaccine and therapeutic development are predominantly focused on the essential virus-encoded Spike, main protease, and RNA-dependent RNA polymerase proteins. These targets have well characterized functions and, particularly for Spike, evidence for neutralizing antibodies in convalescent patient serum. High-resolution structures of these targets, some in complex with drug candidates or neutralizing antibodies, has yielded mechanistic insight into their function and have provided a platform for structure-guided drug design^1–6^. However, expanding of the range of SARS-CoV-2 drug targets may accelerate therapeutic discovery and increase diversity of available drugs to mitigate against the potential evolution of drug-resistant viral strains^7^.

The SARS-CoV-2 genome encodes three putative ion channels (viroporins)^8^, E, 3a, and 8a^9^. Viroporins are generally believed to modify host membrane permeability to promote viral assembly and release, among other functions^8,10^. Ion channels are among the three most commonly targeted protein classes by FDA-approved drugs^11^ and viroporin modulators in particular have had demonstrated therapeutic success, with anti-influenza M2 channel blockers being a well characterized example^12–14^.

In this study, we focus on the SARS-CoV-2 3a channel^15^. The ORF3 genomic region contains coding sequence for multiple open reading frames and exhibits high diversity among coronaviruses compared to neighboring regions^16^. Notably, 3a is highly conserved within the *Betacoronavirus* subgenus *Sarbecovirus* which includes SARS-CoV-1 and related bat coronaviruses that are thought to be the zoonotic source of human-infecting SARS coronaviruses (Fig. S1)^17^. SARS-CoV-1 3a has been reported to form an emodin-sensitive K^+^-permeable cation channel^15,18^ and has been implicated in inflammasome activation^19^ and both apoptotic^20^ and necrotic cell death^21^. In mouse models of SARS-CoV-1 infection, genomic deletion of ORF3a reduced viral titer and morbidity^9^. SARS-CoV-1 3a has therefore been considered a potential target for therapeutics to treat SARS.

3a has three predicted transmembrane helices followed by a cytosolic domain with multiple β-strands per protomer chain^15^. Each of its domains, N-terminal, Transmembrane, and C-terminal, have been proposed to play roles in SARS biology and pathogenesis^15,19^. 3a has been shown to form dimers, tetramers, and potentially higher order oligomers of 31 kDa subunits^15,21^. No structural information exists for 3a proteins nor are there structures of close homologs that could be used to generate structural models, impeding both computational discovery and design of inhibitors as well as a mechanistic understanding of 3a function. To better understand the basis for 3a function, we have determined structures of dimeric and tetrameric SARS-CoV-2 3a in lipid nanodiscs by cryo-electron microscopy (cryo-EM) and characterized 3a channel activity in reconstituted proteoliposomes. This could provide a framework for the design of drugs which target 3a and have the potential to serve as COVID-19 therapeutics.

## Results

Full length SARS-CoV-2 3a was heterologously expressed in *Spodoptera frugiperda* (Sf9) cells with a cleavable C-terminal GFP tag. Whole cell currents recorded from 3a-expressing cells were difficult to distinguish from control cells, likely because the majority of 3a protein is present in intracellular membranes. To better assess 3a channel function, we purified 3a in detergent, reconstituted it into phosphatidylcholine lipids, and recorded currents across excised patches pulled from proteoliposome blisters.

**Figure 1 -:**
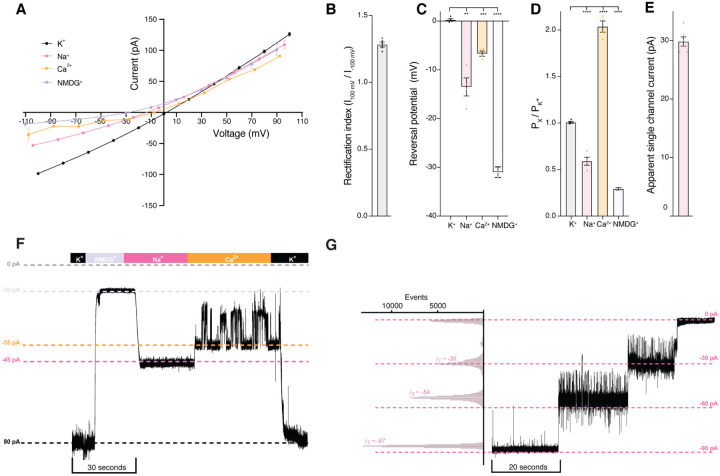
Function of purified and reconstituted SARS-CoV-2 3a (A) Current-voltage relationship from a 3a-proteoliposome patch. Pipette solution was 150 mM K^+^ and external solution was K^+^ (black), Na^+^ (pink), Ca^2+^ (orange), or NMDG^+^ (blue) (mean ± s.e.m., n=4–6 patches). (B) Rectification index (I_100_ mV / I_100_ mV) in symmetrical K^+^. (C) Reversal potential from (A). (D) Permeability ratios $\left({\text{P}}_{\text{X}}/{\text{P}}_{{\text{K}}^{+}}\right)$ calculated from reversal potential shifts in (C). (E) Apparent single channel current at −80 mV with K^+^ pipette and Na^+^ bath solutions. (F) Gap-free current recording held at −80 mV during bath solution exchanges indicated in the bar above the current trace. (G) Gap-free current recording held at −80 mV with K^+^ pipette and Na^+^ bath solutions (right) and corresponding current histogram (left). Differences assessed with a one-way ANOVA with Dunnett correction for multiple comparisons, **p<0.01, *** p<0.001, ****p<0.0001.

3a-containing patches generated currents with modest outward rectification in symmetric [K^+^] (rectification index = 1.28±0.02, mean ±s.e.m. (n=5)), consistent with preferential sidedness of rectifying channels in the membrane after reconstitution (Fig. 1A,B). We evaluated selectivity of 3a for different cations by replacing the K^+^- containing bath solution with solutions containing Na^+^, NMDG^+^, or Ca^2+^. Solution exchange resulted in reversal potential shifts from 0.3±0.3 mV in K^+^ to −6.7±0.5 mV in Ca^2+^, −13.5±1.8 mV in Na^+^, and −31.0±1.1 mV in NMDG^+^ (Fig. 1A,C). These shifts correspond to the following permeability ratios (P /P 2+ X K^+^): Ca (2.04±0.06) > K^+^ (1.0) > Na^+^ (0.59±0.04) > NMDG^+^ (0.29±0.01) (Fig. 1D). We conclude that SARS-CoV-2 3a is a cation channel with modest selectivity for Ca^2+^ and K^+^ over Na^+^.

In K^+^- and Na^+^- containing solutions, channels remained open for long durations with infrequent closures. In contrast, in Ca^2+^-containing solutions, channels exhibited “flickery” behavior with frequent transitions to closed or subconductance states (Fig. 1F). Contrary to previous studies of SARS-CoV-1 3a in cells, SARS-CoV-2 3a activity was not inhibited by Ba^2+^ or by the small molecule emodin^15,18^ (Fig. S2E–H). Alkaline pH modestly increased channel activity (by 31 ± 2% at −80 mV in symmetric K^+^, Fig. S2I,J). Gating was not observed in response to acidic pH (Fig. S2J). After a variable amount of time (on the order of minutes), we observed an increase in “flickery” closures followed by loss of channel activity in each patch, perhaps as a result of channel inactivation (Fig. 1G). The basis for this loss of channel activity remains to be determined. Current reduction occurred in discrete steps of 30.0 ± 0.8 pA from which we estimate the single channel conductance of 3a to be 375 pS at −80 mV under bi-ionic conditions with K^+^ in the pipette and Na^+^ in the bath (Fig. 1E,G).

Purification of 3a in detergent resulted in two species separable by gel filtration (Fig. S3). A majority of 3a runs at a position consistent with a dimer of 62 kDa (Fig. S3A,C,E) and ~10–30% runs as a 124 kDa tetramer (Fig. S3). A similar degree of tetramer formation was observed at low concentrations of 3a by florescence size-exclusion chromatography, indicative of a biochemically stable species rather than concentration-dependent nonspecific aggregation (Fig. S3E). These data are consistent with previous reports of dimeric and tetrameric SARS-CoV-1 3a observed by western blot^15,21^.

**Figure 2 -:**
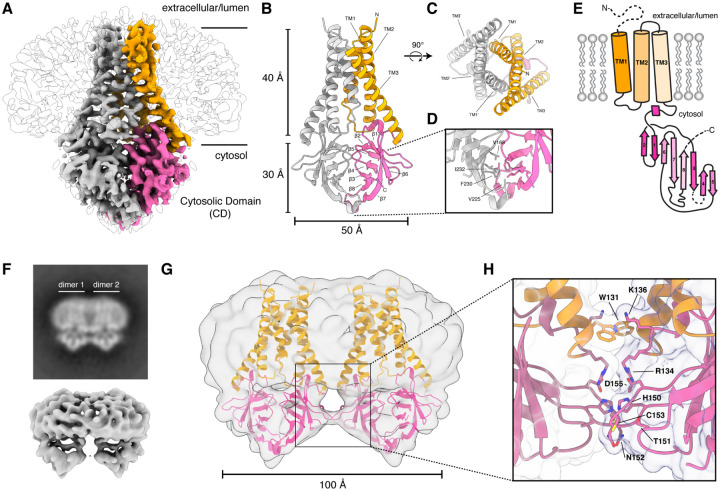
Structure of 3a in lipid nanodiscs (A) Cryo-EM map of the 3a dimer in MSP1E3D1 nanodiscs at 2.9 Å nominal resolution viewed from the membrane plane. One subunit is colored gray and the second subunit is colored with transmembrane region orange and cytosolic domain (CD) pink. Density from the nanodisc is drawn transparent. (B,C) Model of dimeric 3a viewed (B) from the membrane (as in (A)) and (C) from the extracellular or lumenal side. (D) Zoomed in view of the interaction between subunits in the CD with residues forming the hydrophobic core indicated. (E) Schematic of a 3a monomer. Secondary structure elements are indicated and unmodeled termini and a 5 amino acid β3-β4 loop are shown with dashed lines. (F) Two-dimensional class average of tetrameric 3a in MSP1E3D1 lipid nanodiscs (above) and cryo-EM map at 6.5 Å nominal resolution (lower). (G) Two copies of the dimeric 3a structure rigid-body docked into the tetrameric 3a cryo-EM map. (H) Zoomed in view of the interface between two dimers with residues positioned to make contacts indicated.

We separately reconstituted dimeric and tetrameric SARS-CoV-2 3a into nanodiscs made from the scaffold protein MSP1E3D1 and a mixture of POPC, DOPE, and POPS lipids and determined their structures by cryo-EM (Fig. 2A–H, S3B,D, S4–6, S10). We also determined the structure of dimeric 3a in the presence of 100 μM emodin to 3.7 Å, but observed no significant structural changes from dimeric apo 3a or any indication of bound emodin (Fig. S7–9). This is consistent with the lack of emodin inhibition observed in proteoliposome recordings (Fig. S2G,H). The final dimeric reconstruction (with C2 symmetry applied) had an overall resolution of 2.9 Å and permitted *de novo* modeling of 195 of the 275 amino acids per protomer chain (Fig. 2B). The N-terminus (amino acids 1–39), C-terminus (amino acids 239–275), and a short cytoplasmic loop (amino acids 175–180) are either not observed or weakly resolved in the density map, presumably due to conformational differences between particles or because they are disordered.

3a adopts a fold that is, to our knowledge, novel among available protein structures. Querying the protein structure database for structural homologs with Dali returned only weak hits for fragments of 3a domains^22^. Viewed from the membrane plane, 3a is approximately 70 Å tall with a 40 Å high transmembrane region and a cytosolic domain (CD) extending 30 Å out of the membrane (Fig. 2B). The transmembrane region is composed of three helices per protomer. The N-termini are oriented on the extracellular or lumenal side and C-termini on the cytosolic side of the bilayer. 3a is thus a Class IIIA viroporin according to the classification system of Nieva et al^10^. Viewed from the extracellular side, the transmembrane helices (TMs) trace the circumference of an ellipse with TM1–3 from one protomer followed by TM1–3 of the second protomer in a clockwise order (Fig. 2C). TM1s and TM2s pack against each other across the elliptical minor axis with TM3s positioned at the major axis vertices. TM1-TM2 and TM2-TM3 are joined by short intracellular and extracellular linkers, respectively.

The transmembrane region connects to the CD through a turn-helix-turn motif following TM3. Each protomer chain forms a pair of opposing β-sheets packed against one another in an eight stranded β-sandwich (Fig. 2B,D). The outer sheet is formed by strands β1, β2, β6 and the N-terminal half of β7. The inner sheet is formed by strands β3, β4, β5, β8, and C-terminal half of β7. The inner sheets from each protomer interact through a large (~940 Å^2^ of buried surface area per chain) and highly complementary interface with residues V168, V225, F230, and I232 forming a continuous buried hydrophobic core (Fig. 2D). The interaction between β-sandwiches from each protomer thus forms a strong and stable link between monomers in the dimer.

Two-dimensional class averages of tetrameric 3a show a side by side arrangement of two dimers with well-separated TMs and close juxtaposition of CDs (Fig. 2F). Our tetrameric 3a reconstructions had lower final resolutions (~6.5 Å) than dimeric 3a (Fig. 2F, S10). However, the tetrameric map was sufficiently featured in the CDs to enable rigid-body docking of two copies of the 3a dimer model (Fig. 2G). The best fit models show that TM3-CTD linkers and β1-β2 linkers from neighboring dimers form a continuous interface (~300 Å^2^ buried surface area per dimer). While the exact positions of side chains cannot be determined at this resolution, residues W131, R134, K136, H150, T151, N152, C153, and D155 are poised to form a network of hydrophobic, polar, and electrostatic interactions which could mediate tetramerization (Fig. 2H).

Tetramerization of SARS-CoV-1 3a observed by western blot was abolished by reducing agents and a C133A mutation resulted in the loss of tetramerization, membrane localization, and whole-cell currents^15^. However, expression of the SARS-CoV-1 C133A mutant was also dramatically reduced, so it may be that these results are a consequence of destabilizing 3a^15^. In SARS-CoV-2 3a, C133 is located in a notable cysteine-rich pocket adjacent to the tetramerization interface (Fig. S11A). At the base of TM3, C133 projects back towards the top faces of β1 and β2 in close proximity to solvent exposed C148 and buried C157. Due to geometric considerations, we modeled C133 and C157 as reduced sulfhydryls, but note that they are nearly within disulfide-bonding distance (Cα distance 6.9 Å) (Fig. S11A). While it is unlikely a single conserved disulfide mediates tetramerization in SARS-CoV-1 3a and SARS-CoV-2 3a without significant rearrangement of this region, it may be that disruption of this cysteine-rich pocket with cysteine modifying agents or mutations disfavors 3a oligomerization.

**Figure 3 -:**
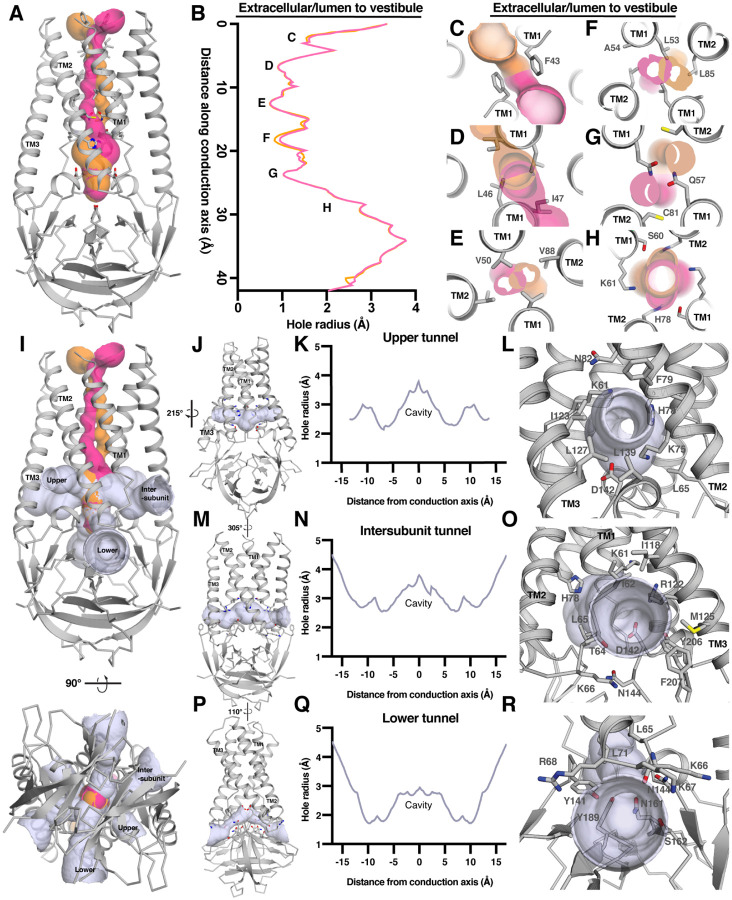
The 3a channel pore (A) View of a 3a dimer from the membrane plane with the bifurcated pore connecting the exterior solution to the channel cavity colored in pink and orange. (B) Radii of the pores shown in (A) as a function of distance along the conduction axis. (C-H) Zoomed in views from the external side looking down the pore of six constrictions labeled in (B). (I) View of 3a dimer (upper) from the membrane as in (A) and (lower) from the cytosol with the three pairs of tunnels shown in blue. The upper tunnels open to the surrounding lipid bilayer, the intersubunit tunnels open to the membrane-cytosol interface, and the lower tunnels open to the cytosol. (J) View from the membrane of the upper tunnels. (K) Radius of the upper tunnel as a function of distance from the conduction axis. (L) View from the cytosol into the upper tunnel with residues contributing to the tunnel surface indicated. (M-O) Same as (J-L) for the intersubunit tunnels. (P-R) Same as (J-L) for the lower tunnels.

Analysis of possible conduction pathways through 3a reveals a narrow, bifurcated pore which stretches through the outer half of the TM region and is connected to a large and polar cavity open to the cytosol (Fig. 3). The pore has a series of six constrictions and narrows to ~1 Å in radius, too small for the conduction of permeant cations. We therefore conclude this 3a structure represents either a closed or inactivated channel conformation.

From the extracellular side, the first four constrictions are hydrophobic (Fig. 3A–F). Opposing F43 residues at the top of TM1 create two paths into the channel which taper to ~1.5 Å in radius. The paths then merge and go through a series of tight (~1 Å radius) constrictions lined by L46 and I47, V50 and V58, and finally L53 and L85. The final two constrictions are polar (Fig. 3G,H). The close juxtaposition of Q57s from opposing TM1s splits the pore into two paths of ~1 Å radius lined by C81s from TM2s. The final and widest constriction (~2 Å radius) before the pore opens to the cavity is lined by S60 and K61 from TM1 and H78 from TM2. Opening the channel would require conformational changes in TM1 and/or TM2 to both expand the hydrophobic constrictions and displace Q57 at the inner hydrophilic constriction.

The polar cavity within the inner half of the TM region is continuous with the cytosol and surrounding bilayer through three pairs of openings: the upper, intersubunit, and lower tunnels (Fig. 3I–R). The upper tunnels are formed between TM2 and TM3 within each protomer and narrow to ~2 Å in radius (Fig. 3J–L). Judging by the relative position of the nanodisc in the EM maps and hydrophobic character of the upper tunnel exterior, in a cell membrane they likely open to the surrounding lipid bilayer. The intersubunit tunnels run between TM1 and TM3 from different protomers, just above the CD, and narrow to ~2.5 Å in radius (Fig. 3M–O). With a modest expansion, the intersubunit tunnels could permit even large cations like NMDG to access the channel cavity. The intersubunit tunnels open to the membrane-cytosol interface. Tubular shaped-densities are present in both the upper and intersubunit tunnels which are consistent with lipid acyl chains, but are not sufficiently featured to confidently model as such (Fig. S11B). The lower tunnels run underneath the TM1-TM2 linker and above the CD and narrow to ~2 Å in radius (Fig. 3P–R). The lower tunnels open well into the cytosol and are open paths for ion movement between the cell interior and channel cavity.

**Figure 4 -:**
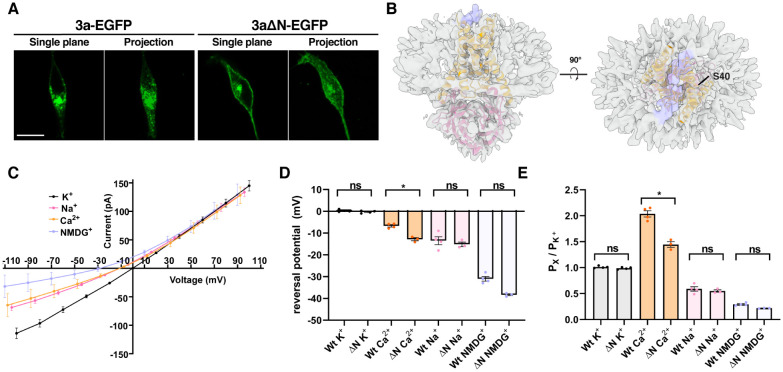
The 3a N-terminus is involved in subcellular localization (A) 3a-GFP fluorescence localization in HEK cells transfected with 3a-EGFP or 3aΔN-EGFP. Single plane and brightest-point projection are displayed for each. Scale bar, 10 μm. (B) Dimeric 3a cryo-EM density map (gray) with unmodeled extended density above the mouth of the pore that may correspond to the N-terminal regions colored blue. A 3a dimer model is drawn pink inside the density. (C) Current-voltage relationship from a 3aΔN-proteoliposome patch. Pipette solution was 150 mM K^+^ and external solution was K^+^ (black), Na^+^ (pink), Ca^2+^ (orange), or NMDG^+^ (blue) (mean ± s.e.m., n=3–4 patches). (D) Reversal potential from (C). (E) Permeability ratios $\left({\text{P}}_{\text{X}}/{\text{P}}_{{\text{K}}^{+}}\right)$ calculated from reversal potential shifts in (D). Data for wild-type are replicated from Figure 1 for comparison. Differences assessed with a one-way ANOVA with Dunnett correction for multiple comparisons, *p=0.01, ns=not significant; p>0.05.

The N-terminal ~41 residues of each chain constitute the majority of the extracellularly or lumenally exposed 3a protein and thus could be involved in retention of 3a to internal membranes in cultured cells. To test this, we generated an N-terminal deletion construct lacking the first 41 amino acids (3aΔN) and compared its localization to wild-type 3a in HEK cells. Indeed, 3aΔN-EGFP shows reduced localization to internal membranes and bright foci and increased plasma membrane expression (Fig. 4A, S12). While we were unable to model the N-terminal 39 residues of 3a, we note an unassigned density feature in the cryo-EM maps that stretches between subunits just above the extracellular entrance to the pore that could correspond to a portion of these unmodeled N-terminal residues (Fig. 4B). We speculated that if the N-terminal region was stably positioned above the mouth of the pore it could influence channel properties. 3aΔN was therefore purified, reconstituted into proteoliposomes and compared to wild type 3a in patch recordings. Aside from a modest decrease in the relative permeability of Ca^2+^ to K^+^, no significant differences were observed in 3aΔN properties compared to wild-type 3a (Fig. 4C–E, S13). These results are consistent with the N-terminal region of 3a being a determinant of its subcellular localization without influencing channel properties.

**Figure 5 -:**
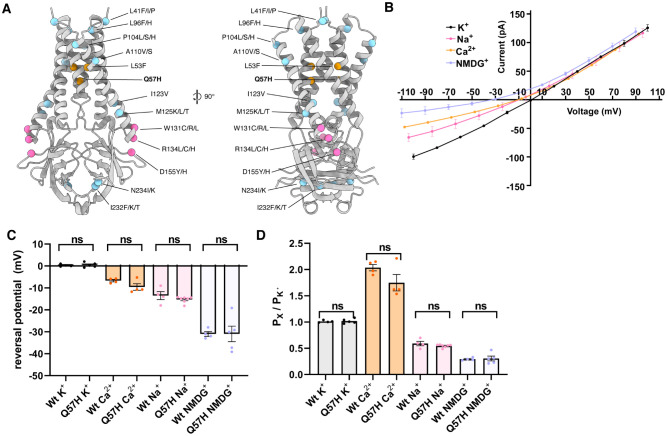
Structural and functional analysis of the common 3a variant Q57H (A) View of 3a from the membrane plane (left) and rotated 90° about the conduction axis (right) with the positions of known coding variants indicated. Variants in the 3a pore (Q57H and L53F) are colored orange, variants at the tetramerization interface (W131C/R/L, R134L/C/H, and D155Y/H) are colored pink, and variants unlikely to impact structure or function of 3a are colored blue. (B) Current-voltage relationship from a 3a Q57H-proteoliposome patch. Pipette solution was 150 mM K^+^ and external solution was K^+^ (black), Na^+^ (pink), Ca^2+^ (orange), or NMDG^+^ (blue) (mean ± s.e.m., n=4–7 patches). (C) Reversal potential from (B). (D) Permeability ratios $\left({\text{P}}_{\text{X}}/{\text{P}}_{{\text{K}}^{+}}\right)$ calculated from reversal potential shifts in (C). Data for wild-type are replicated from Figure 1 for comparison. Differences assessed with a one-way ANOVA with Dunnett correction for multiple comparisons, ns=not significant; p>0.05.

Over forty thousand SARS-CoV-2 genomes have been sequenced to date and analyses of mutations across time and geography have identified a large number of coding variants, some of which may have experienced selective pressure during viral evolution^45^. To date, mutations that result in amino acid changes at 17 residues in 3a have been observed in different SARS-CoV-2 genomes^23,45^. Thirteen of these residues are represented with colored spheres overlaid on the 3a structure in Figure 5A (the remaining four positions: K16N/L, P36L, P240L/S/H, and P258L are unresolved in our structure). The most prevalent is a Q57H variant found in ~25% of sequenced viruses, yet not observed in the earliest sequences or in related bat coronaviruses. Strikingly, as described above, Q57 forms the major hydrophilic constriction in the 3a pore (Fig. 3G, 5A). We asked whether this mutation had functional consequences by purifying 3a-Q57H, reconstituting it into proteoliposomes, and comparing channel activity to wild-type (Fig. 5B–D). No significant differences were observed in 3a-Q57H properties compared to wild-type 3a (Fig. 5B–D, S13A–C). We conclude that the presence of a histidine at this position does not influence channel properties. The remaining variants are much less common, being observed in <0.1% of sequenced viruses to date. Nine of these are unlikely to impact 3a structure or function as they are located in loops, lipid facing positions on TMs, and/or are conservative in nature (L41F/I/P, L53F, L96F/H, P104L/S/H, A110V/S, I123V, M125K/L/T, I232F/K/T and N234I/K). The remaining three are at the tetramerization interface (W131C/R/L, R134L/C/H, D155Y/H) (Fig. 2H, 5A). Whether these influence oligomerization or channel function remains to be determined.

3a is very well conserved in the *Betacoronavirus* subgenus *Sarbecovirus* that includes SARS-CoV-1 and SARS-CoV-2 (Fig. S1). Structurally related proteins have not been identified in other coronaviruses (including the other five species known infect humans: MERS-CoV, HCoV-NL63, HCoV-229E, HCoV-HKU1, and HCoV-OC43) by sequence homology. We asked whether we could identify more distant homologs using structure prediction algorithms and the SARS-CoV-2 3a structure. *Coronaviridae* are classified into 4 genera: *Alphacoronavirus*, *Betacoronavirus*, *Gammacoronavirus*, and *Deltacoronavirus*. 3a homologs were not detected in any *Gammacoronavirus* or *Deltacoronavirus* species or in the *Betacoronavirus* subgenus *Embecovirus* (which includes HCoV-HKU1 and HCoV-OC43). Distant homology to the CD was identified in the membrane protein ORF5 found in *Betacoronavirus* subgenus *Merbecovirus* species including MERS-CoV. In contrast, high confidence structural homologs were predicted in all remaining *Betacoronavirus* subgenera in proteins annotated ORF3 or NS3 and in all *Alphacoronavirus* subgenera in proteins annotated ORF3, NS3, ORF4, or NSP3B (including in HCoV-229E and HCoV-NL63 (Fig. S14)). Several of these homologs have been previously demonstrated to have ion channel activity^24–26^. Strikingly, all coronaviruses with 3a structural homologs are derived from the bat gene pool, while all those without 3a structural homologs derive from rodent, avian, or pig gene pools (Table 2). This suggests coevolution of 3a with coronaviruses that have bats as their principal reservoir and may reflect a unique aspect of bat coronavirus biology. The true extent of 3a structural and functional conservation awaits further experimental confirmation. Still, this analysis suggests 3a or 3a-like proteins are more broadly present in coronaviruses than previously recognized and antiviral drugs targeting these proteins could potentially treat diseases associated with multiple known human coronavirus. Further experiments that resolve the role of 3a in coronavirus biology and pathology could aid in the development of therapeutics targeting 3a channels.

## Methods

### Cloning and protein expression

The coding sequence for the 3a protein from SARS-Cov-2 was codon optimized for Spodoptera frugiperda (Sf9 cells) and synthesized (IDT, Newark, NJ). The sequence was then cloned into a custom vector based on the pACEBAC1 backbone (MultiBac; Geneva Biotech, Geneva, Switzerland) with an added C-terminal PreScission protease (PPX) cleavage site, linker sequence, super-folder GFP (sfGFP) and 7xHis tag, generating a construct for expression of 3a-SNS-LEVLFQGP-SRGGSGAAAGSGSGS-sfGFP-GSS-7xHis. Mutants and truncation were also introduced into this construct using PCR. MultiBac cells were used to generate a Bacmid according to manufacturer’s instructions. Sf9 cells were cultured in ESF 921 medium (Expression Systems, Davis, CA) and P1 virus was generated from cells transfected with Escort IV reagent (MillaporeSigma, Burlington, MA) according to manufacturer’s instructions. P2 virus was then generated by infecting cells at 2 million cells/mL with P1 virus at a MOI ~0.1, with infection monitored by fluorescence and harvested at 72 hours. P3 virus was generated in a similar manner to expand the viral stock. The P3 viral stock was then used to infect Sf9 cells at 4 million cells/mL at a MOI ~2–5. At 72 hours, infected cells containing expressed 3a-sfGFP protein were harvested by centrifugation at 2500 x g for 10 minutes and frozen at −80°C.

### Protein purification

For preparation of the 3a dimer and mutant constructs, infected Sf9 cells from 1 L of culture (~15–20 mL of cell pellet) were thawed in 100 mL of Lysis Buffer containing 50 mM Tris, 150 mM KCl, 1mM EDTA pH 8. Protease inhibitors (Final Concentrations: E64 (1 μM), Pepstatin A (1 μg/mL), Soy Trypsin Inhibitor (10 μg/mL), Benzimidine (1 mM), Aprotinin (1 μg/mL), Leupeptin (1μg/mL), AEBSF (1mM), and PMSF (1mM)) were added to the lysis buffer immediately before use. Benzonase (4 μl) was added after the cell pellet thawed. Cells were then lysed by sonication and centrifuged at 150,000 x g for 45 minutes. The supernatant was discarded and residual nucleic acid was removed from the top of the membrane pellet using DPBS. Membrane pellets were scooped into a dounce homogenizer containing Extraction Buffer (50 mM Tris, 150 mM KCl, 1 mM EDTA, 1% n-Dodecyl-β-D-Maltopyranoside (DDM, Anatrace, Maumee, OH), pH 8). A 10% stock solution of DDM was dissolved and clarified by bath sonication in 200 mM Tris pH 8 prior to addition to buffer to the indicated final concentration. Membrane pellets were then homogenized in Extraction Buffer and this mixture (150 mL final volume) was gently stirred at 4°C for 1 hour. The extraction mixture was centrifuged at 33,000 x g for 45 minutes and the supernatant, containing solubilized membrane protein, was bound to 4 mL of Sepharose resin coupled to anti-GFP nanobody for 1 hour at 4°C. The resin was then collected in a column and washed with 10 mL of Buffer 1 (20 mM HEPES, 150 mM KCl, 1 mM EDTA, 0.025% DDM, pH 7.4), 40 mL of Buffer 2 (20 mM HEPES, 500 mM KCl, 1 mM EDTA, 0.025% DDM, pH 7.4), and 10 mL of Buffer 1. The resin was then resuspended in 6 mL of Buffer 1 with 0.5 mg of PPX protease and rocked gently in the capped column for 2 hours. Cleaved 3a protein was then eluted with an additional 8 mL of Wash Buffer, spin concentrated to ~500 μl with Amicon Ultra spin concentrator 10 kDa cutoff (Millipore), and then loaded onto a Superdex 200 increase column (GE Healthcare, Chicago, IL) on an NGC system (Bio-Rad, Hercules, CA) equilibrated in Buffer 1. Peak fractions containing 3a channel were then collected and spin concentrated prior to incorporation into proteoliposomes or nanodiscs. For the tetramer, the preparation was carried out in a similar manner, except with overnight protease cleavage and collection of a peak of larger hydrodynamic radius (see Fig. S3).

### Proteoliposome formation

For proteoliposome patching experiments, we incorporated protein into lipid and generated proteoliposome blisters for patch recordings using dehydration and rehydration as described previously with the following modifications^27^. 3a dimer was first purified into Buffer 1. Protein was then exchanged into lipid with the addition of Biobeads SM2 and an overnight incubation at a protein:lipid ratio of 1:10 (corresponding to 0.5 mg purified 3a dimer and 5 mg of cleared Soybean L-α-phosphatidylcholine (Soy PC, MillaporeSigma, Burlington, MA) in DR Buffer (5 mM HEPES, 200 mM KCl, pH7.2).

### Electrophysiology

All electrophysiology recordings were made from 3a-reconstituted Soy PC proteoliposomes. Patches formed in an inside-out configuration and were quickly (within 5–10 seconds) transferred to a solution exchange chamber. Recordings were made at room temperature using Clampex 10.7 data acquisition software with an Axopatch 200B Patch Clamp amplifier and Digidata 1550B digitizer (Molecular Devices) at a bandwidth of 1 kHz and digitized at 500 kHz. A pressure clamp (ALA Scientific) was used to form seals. Potassium pipette and bath solution was 5 mM HEPES pH 7.2, 150 mM KCl, 5 mM EGTA, 1 mM MgCl2. Sodium bath solution was 5 mM HEPES pH 7.2, 150 mM NaCl, 1 mM MgCl_2_, 1 mM CaCl_2_. NaCl in the bath solution was substituted for 150 mM NMDG-Cl or 75 mM CaCl_2_ for permeability experiments. Borosilicate glass pipettes were pulled and polished to a resistance of 2–5 MΩ when filled with pipette solution. For cation permeability experiments, liquid junction potentials were calculated and data were corrected offline. For current-voltage plots, the following voltage protocol was applied: V_hold_= 0 mV mV; V_test_= −100 to +100 mV, Δ20 mV, t_test_ = 1 second. Currents from each patch correspond to mean values during the step to the indicated voltage.

Permeability ratios were calculated according to Goldman-Hodgkin-Katz relationship. For mono-valent cations, permeability ratios were calculated as ${\text{P}}_{\text{X}+}/{\text{P}}_{{\text{K}}^{+}}\;=\;\text{exp}\left(\Delta {\text{V}}_{\text{rev}}\text{F}/\text{RT}\right)$. For divalent cations, permeability ratios were calculated as: ${\text{P}}_{{\text{X}}^{2+}}/{\text{P}}_{{\text{K}}^{+}}\;=\;\alpha_{{\text{K}}^{+}}\left[{\text{K}}^{+}\right]\text{exp}\left(\Delta {\text{V}}_{\text{rev}}\text{F}/\text{RT}\right)\left(1\;+\;\text{exp}\left(\Delta {\text{V}}_{\text{rev}}\text{F}/\text{RT}\right)\right)/4\alpha_{{\text{X}}^{2+}}\left[{\text{X}}^{2+}\right]$ Where V_rev_ is the reversal potential, F is Faraday’s constant, R is the universal gas constant, and T is absolute temperature (where RT/F = 25.2 mV at room temperature), and α is the ion activity coefficient (assumed to be 0.75 for K^+^ and 0.25 for Ca^2+^).

### Nanodisc formation

Freshly purified 3a dimer in Buffer 1 was reconstituted into MSP1E3D1 nanodiscs with a mixture of lipids (DOPE:POPS:POPC at a 2:1:1 mass ratio, Avanti, Alabaster, Alabama) at a final molar ratio of 1:4:400 (Monomer Ratio: 3a, MSP1E3D1, lipid mixture). First, 20 mM solubilized lipid in Nanodisc Formation Buffer (20 mM HEPES, 150 mM KCl, 1 mM EDTA pH 7.4) was mixed with additional DDM detergent and 3a protein. This solution was mixed at 4°C for 30 minutes before addition of purified MSP1E3D1. This addition brought the final concentrations to approximately 15 μM 3a, 60 μM MSP1E3D1, 6 mM lipid mix, and 10 mM DDM in Nanodisc Formation Buffer. The solution with MSP1E3D1 was mixed at 4°C for 10 minutes before addition of 200 mg of Biobeads SM2 (Bio-Rad, Hercules, CA). Biobeads (washed into methanol, water, and then Nanodisc Formation Buffer) were weighed after liquid was removed by pipetting (damp weight). This mix was incubated at 4°C for 30 minutes before addition of another 200 mg of Biobeads (for a total 400 mg of Biobeads per 0.5 mL reaction). This final mixture was then gently tumbled at 4°C overnight (~12 hours). Supernatant was cleared of beads by letting large beads settle and carefully removing liquid with a pipette. Sample was spun for 10 minutes at 21,000 x g before loading onto a Superdex 200 increase column in 20 mM HEPES, 150 mM KCl, pH 7.4. Peak fractions corresponding to 3a protein in MSP1E3D1 were collected, 10 kDa cutoff spin concentrated and used for grid preparation. MSP1E3D1 was prepared as described^28^ without cleavage of the His-tag. Tetrameric 3a in nanodiscs was prepared similarly, except with a ratio of 1:2:200 (Monomer Ratio: 3a, MSP1E3D1, lipid mixture).

### Grid preparation

Dimeric 3a in MSP1E3D1 was prepared at final concentration of 1.1 mg/mL. For the sample with emodin (MillaporeSigma, Burlington, MA, Catalog E7881), a stock solution of 50 mM emodin in DMSO added to protein sample for final concentrations of 1.1 mg/mL 3a and 100 μM emodin and 1% DMSO. Concentrated sample was cleared by a 10 minute 21,000 x g spin at 4°C prior to grid making. For freezing grids, a 3 μl drop of protein was applied to freshly glow discharged Holey Carbon, 300 mesh R 1.2/1.3 gold grids (Quantifoil, Großlöbichau, Germany). A FEI Vitrobot Mark IV (ThermoFisher Scientific) was used with 4°C, 100% humidity, 1 blot force, a wait time of ~5 seconds, and a 3 second blot time, before plunge freezing in liquid ethane. Grids were then clipped and used for data collection. Tetrameric 3a in MSP1E3D1 was frozen at 0.7 mg/mL with the same grid preparation.

### Cryo-EM data acquisition

Grids were clipped and transferred to a FEI Talos Arctica electron microscope operated at 200 kV. Fifty frame movies were recorded on a Gatan K3 Summit direct electron detector in super-resolution counting mode with pixel size of 0.5685 Å. For the apo 3a dataset, the electron dose was 9.528 e^−^ Å^2^ s^−1^ and 10.135 e^−^ Å^2^ s^−1^ and total dose was 50.02 e^−^ Å^2^ and 53.72 e^−^ Å^2^ in the first set (1–2007) and second set (2008–6309) of movies respectively. The two different doses are the result of needing to restart the electron gun during collection. For the 3a with added emodin dataset, the electron dose was 8.991 e^−^ Å^2^ s^−1^ and total dose was 47.21 e^−^ Å^2^. For the 3a tetramer, the electron dose was 8.841 e^−^ Å^2^ s^−1^ and total dose was 49.95 e^−^ Å^2^. Nine movies were collected around a central hole position with image shift and defocus was varied from −0.6 to −2.0 μm through SerialEM^29^. See Table 1 for data collection statistics.

### Cryo-EM data processing

For the apo 3a dimer, motion-correction and dose-weighting were performed on all 6,309 movies using RELION 3.1’s implementation of MotionCor2, and 2x “binned” to 1.137 Å per pixel^30–32^. CTFFIND-4.1 was used to estimate the contrast transfer function (CTF) parameters^33^. Micrographs were then manually sorted to eliminate subjectively bad micrographs, such as empty or contaminated holes, resulting in 3,611 good micrographs. Additionally, micrographs with a CTF maximum resolution lower than 4 Å were discarded, resulting in 2,595 remaining micrographs. Template-free auto-picking of particles was performed with RELION3.1’s Laplacian-of-Gaussian (LoG) filter yielding an initial set of particles. This initial set of particles were iteratively classified to generate templates, which were subsequently used to template-based auto-pick 1,750,730 particles.

Template picked particles were iteratively 2D-classified in RELION3.1 and then in cryoSPARC v2^34^, resulting in 820,543 particles. These particles were subsequently 3D-classified in cryoSPARC v2 with iterative ab-initio and heterogeneous refinement jobs. The resulting maps were visually evaluated with regard to the transmembrane domain density. A set of 86,479 particles were identified, polished in RELION3.1 and refined in cryoSPARC v2 with subsequent homogeneous and non-uniform refinement^35^ jobs (maps were low-pass filtered to an initial resolution where TM density was still visible (6–9 Å), and the dynamic mask was tightened with the near (2–5 Å) and far (3–9 Å) parameters), yielding a map with overall resolution of 3.6 Å. UCSF pyem tools were used to covert data from cryoSPARC to RELION format^44^.

From this set of 86,479 particles, 2D-classification was performed in RELION3.1 to identify a set of particles with subjectively equal view distribution. From the resulting set, 1,000 particles were randomly sampled and their coordinates used for training in the Topaz particle-picking pipeline^36^. Training, picking, and extraction were performed independently on each subset of the micrographs. 4,134,279 total particles were extracted in RELION3.1 with a box size of 256 pixels and “binned” 4x to 4.548 Å/pixel. These particles were then iteratively 2D-classified in RELION3.1 resulting in 2,674,606 particles which were extracted at 2.274 Å/pixel. 2D-classification was continued in both RELION3.1 and cryoSPARC v2 resulting in 1,429,763 particles. Further classification was performed in cryoSPARC v2 with subsequent ab-initio (4 classes, max resolution 8 Å) and heterogeneous refinement (8 Å initial resolution) jobs. The two best classes were selected and the particles pooled resulting in 743,800 particles which were extracted in RELION3.1 at 1.137 Å/pixel.

Iterative 3D-classification was performed with subsequent ab-initio and heterogeneous refinement jobs as described above. Following each round, 2D classification jobs were used to “rescue” good particles from the worst classes before the next round. After 3 rounds, a final 2D-classificaiton job was used to identify 112,502 particles, which were subsequently pooled with the previous 86,479 RELION3.1 template-picked particles, resulting in 185,871 particles after duplicates (within 100 Å) were removed with RELION3.1.

These particles were then refined with subsequent homogeneous and non-uniform refinement jobs resulting in a map with overall resolution of 3.4 Å. This map was post-processed in RELION3.1 using a mask with a soft edge (5 pixel extension, 7 pixel soft-edge), the output of which was used for Bayesian particle polishing in RELION3.1 (training and polishing were each performed independently on each subset of the micrographs). The resulting “shiny” particles were then refined in cryoSPARC v2 with subsequent homogenous refinement (1 extra pass, 7 Å initial resolution) and non-uniform refinement (C2, 1 extra pass, 9 Å initial resolution) to yield a map with 2.9 Å overall resolution.

For 3a dimer with added 100 μM emodin, initial processing was similar to the dimer without added drug (see Fig. S8). As with the apo 3a dimer, the critical steps included Topaz particle picking, particle clean-up with cryoSPARC v2 ab-initio and heterogeneous refinement, non-uniform refinement with tightened masking, and RELION3.1 Bayesian particle polishing. However, in contrast to the apo dataset, we observed a set of particles that were included in < 4 Å reconstructions that had discontinuous transmembrane domain density. Removal of these particles with RELION3.1 3D classification without angular sampling led to the best map from the emodin-added dataset. We did not see any evidence of bound emodin, but the 1% DMSO added with drug addition may have contributed to subtle map differences (Fig. S8,S9).

For the 3a tetramer, the initial 7,092 micrographs were first cleaned using manual inspection and removal of images with < 4 Å CtfMaxResolution to obtain a set of 4,324 micrographs. Reference particles for Topaz particle picking were generated by first template picking in RELION3.1, followed by 2D classification in both RELION3.1 and cryoSPARC v2, and subsequent ab-initio in cryoSPARC v2. Particles from various views were then selected from iterative RELION3.1 2D classification to create a set of 6,843 particles. Using these coordinates for training, Topaz particle picking was then performed to generate a set of 1,282,913 initial particles. These particles were then cleaned using 2D classification in RELION3.1 and cryoSPARC v2, followed by rounds of cryoSPARC v2 ab-initio and RELION3.1 3D classification. A major hurdle for tetramer processing was obtaining a reconstruction where most particles were properly oriented in the same direction (i.e. CD domains on the same side of the nanodisc as seen in the 2D classes, see Fig. 2F). Substantial, cleanup by 3D-classification was needed to generate a correctly aligned reference map, but this map could then be used as a reference for refinements and classification for larger particle sets. Reconstructions with C1 or C2 symmetry looked similar (see Fig. S10), although no tetramer reconstruction went to high enough resolution to determine symmetry with certainty. Therefore, it is possible that either the tetramer is pseudosymmetric or that different particles have heterogeneous orientations between dimer pairs. For the tetramer, the highest resolution reconstruction came from cryoSPARC v2 non-uniform refinement with a tightened mask, which was subsequently used for dimer-docking and figure preparation.

### Modeling, Refinement, and Analysis

Apo dimeric 3a cryo-EM maps were sharpened using cryoSPARC and were of sufficient quality for de novo model building in Coot^37^. Real space refinement of the models was carried out using Phenix.real_space_refine^38^. Molprobity^39^ was used to evaluate the stereochemistry and geometry of the structure for subsequent rounds of manual adjustment in Coot and refinement in Phenix. Docking of the apo dimeric 3a into the tetrameric 3a cryo-EM map was performed in Phenix using a map in which large empty regions of the nanodisc were erased in Chimera^40^. Similar results were found using maps with only the CDs present. Cavity measurements were made with HOLE^41^ implemented in Coot. Comparisons to the structure database was performed with DALI^22^. Structure prediction was performed with Phyre2^42^. Figures were prepared using PyMOL, Chimera, ChimeraX^43^, Fiji, Prism, GNU Image Manipulation Program, and Adobe Photoshop and Illustrator software.

### Fluorescence Size Exclusion Chromatography (FSEC)

Sf9 cells (~4 million) from the third day of infection were pelleted, frozen, and then thawed into extraction buffer (20mM Tris pH 8, 150 mM KCl, all protease inhibitors used for protein purification, 1 mM EDTA, 1% DDM). Extraction was performed at 4°C for 1 hour and lysate was then pelleted at 21,000 x g at 4°C for 1 hour to clear supernatant. Supernatant was then run on a Superose 6 Increase column with fluorescence detection for GFP into 20 mM HEPES pH 7.4, 150 mM KCl, 0.025% DDM.

### Transfection and Imaging

The constructs for full length 3a and 3aΔN were cloned into a vector with a CMV-promoter and C-terminal EGFP. Constructs (2 μg) were transfected into HEK293 cells on glass coverslips using Fugene HD (Promega, Madison, WI) per manufacturer’s instructions. Two days after transfection cells were washed with DPBS and then fixed in 4% Formaldehyde in DPBS for 10 minutes. Cells were then washed with DPBS before mounting the coverslip with Prolong Glass Antifade with NucBlue (ThermoFisher Scientific) per manufacturer’s instructions. Fluorescent images were collected using a Zeiss LSM 880 NLO AxioExaminer confocal microscope at either 20X (NA 1.0) or 63X oil immersion objective (NA 1.4). The samples were excited with 488nm argon laser and image analysis was performed using ImageJ.

## Supplementary Material

1
