# Supplementary material for: Cryo-EM structure of the SARS-CoV-2 3a ion channel in lipid nanodiscs
Source: bioRxiv. 2021 Jan 26:2020.06.17.156554. Originally published 2020 Jun 18. Preprint. [Version 3] doi: 10.1101/2020.06.17.156554 (PMC7310636; doi:10.1101/2020.06.17.156554)
Supplement: 1 [file NIHPP2020.06.17.156554-supplement-1.pdf]

bioRxiv preprint doi: https://doi.org/10.1101/2020.06.17.156554; this version posted January 26, 2021. The copyright holder for this preprint (which was not certified by peer review) is the author/funder, who has granted bioRxiv a license to display the preprint in perpetuity. It is made available under aCC-BY 4.0 International license.

Figure S1

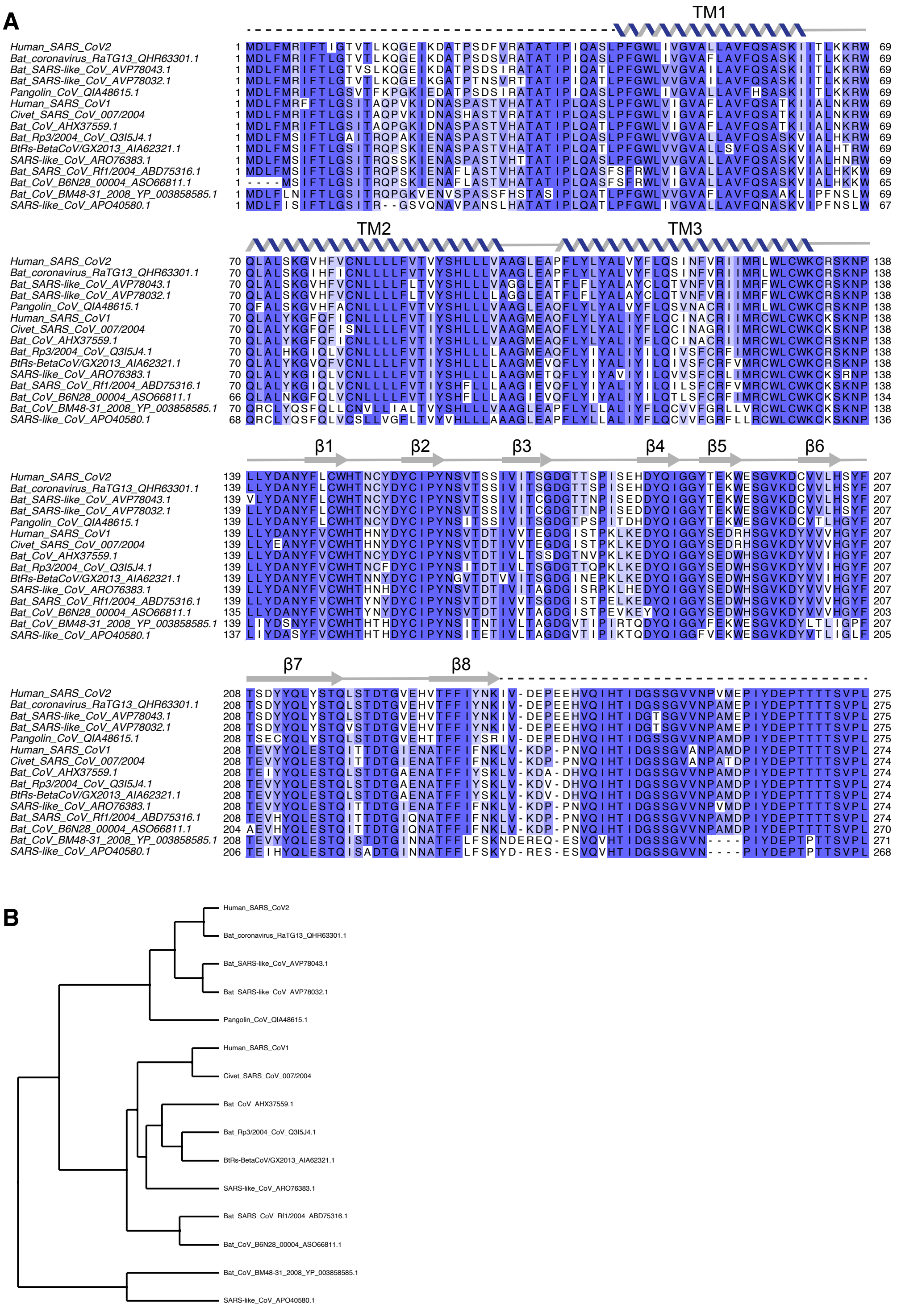

Figure S1 – Sequence alignment of 3a from *Betacoronavirus Sarbecovirus*
(A) Alignment of fifteen 3a protein sequences colored by conservation in a ramp from white (not conserved) to dark blue (highly conserved). Accession numbers are indicated. Sequences were selected to maximize diversity among annotated *Sarbecovirus* 3a proteins. Secondary structure for SARS-CoV-2 is drawn above the sequence with unmodeled sequence drawn as dashed lines. (B) Neighbor-joining tree calculated from the alignment in (A).

bioRxiv preprint doi: https://doi.org/10.1101/2020.06.17.156554; this version posted January 26, 2021. The copyright holder for this preprint (which was not certified by peer review) is the author/funder, who has granted bioRxiv a license to display the preprint in perpetuity. It is made available under aCC-BY 4.0 International license.

Figure S2

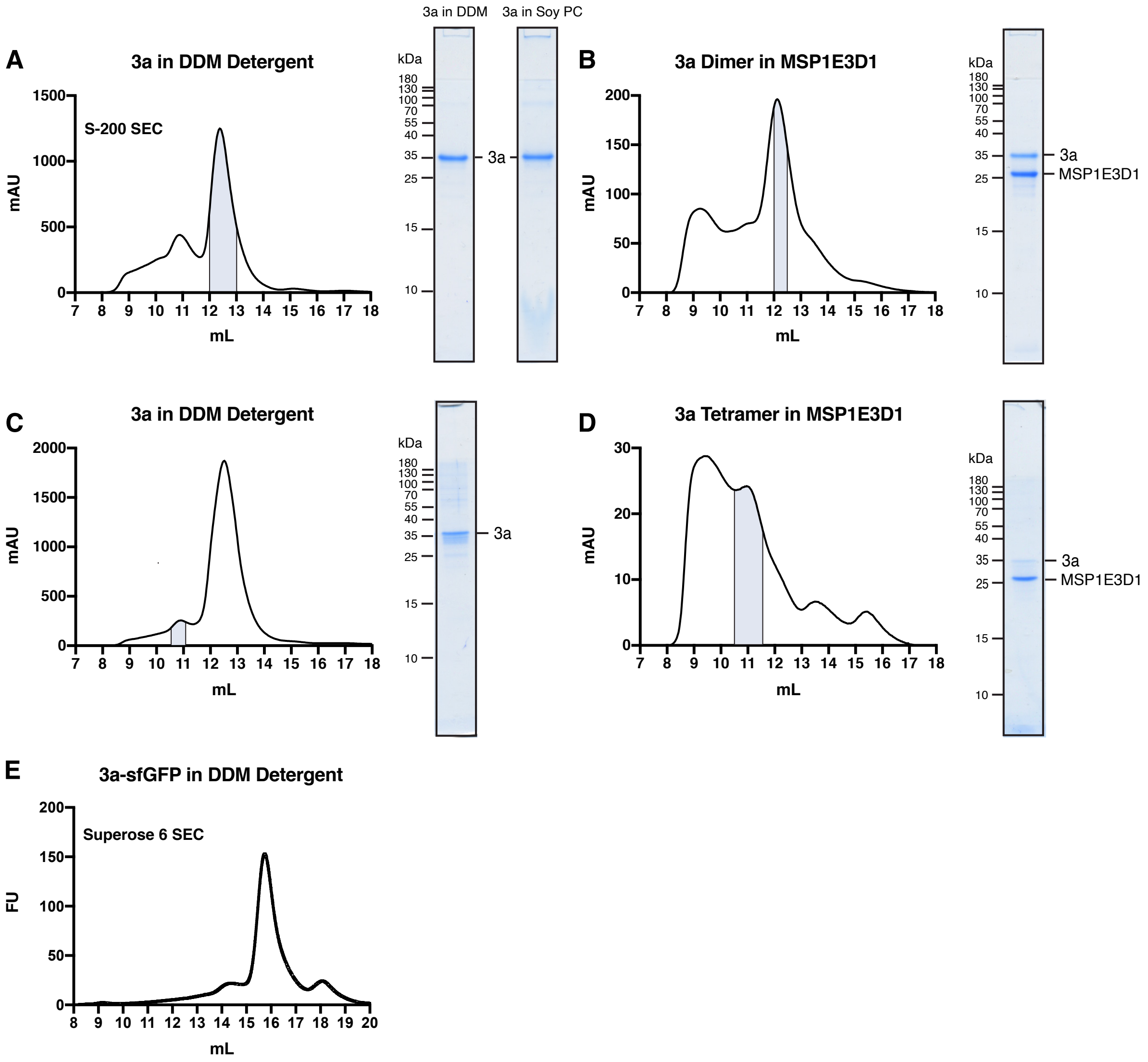

Figure S2 – Purification and reconstitution of 3a

(A) Size exclusion chromatogram of 3a expressed in insect cells and extracted and purified in DDM (left). Pooled fractions corresponding to dimeric 3a are highlighted in blue. Coomassie-stained SDS-PAGE of pooled dimeric 3a-containing fractions (center) and of 3a following reconstitution into PC lipids (right). (B) Size exclusion chromatogram of dimeric 3a reconstituted into MSP1E3D1 lipid nanodiscs (left). Pooled fractions are highlighted blue. (C,D) Same as (A,B), but for tetrameric 3a. (E) GFP fluorescence chromatogram of 3a expressed in SF9 cells and extracted in DDM detergent. Samples were run on a Superose 6 column.

bioRxiv preprint doi: https://doi.org/10.1101/2020.06.17.156554; this version posted January 26, 2021. The copyright holder for this preprint (which was not certified by peer review) is the author/funder, who has granted bioRxiv a license to display the preprint in perpetuity. It is made available under aCC-BY 4.0 International license.

Figure S3

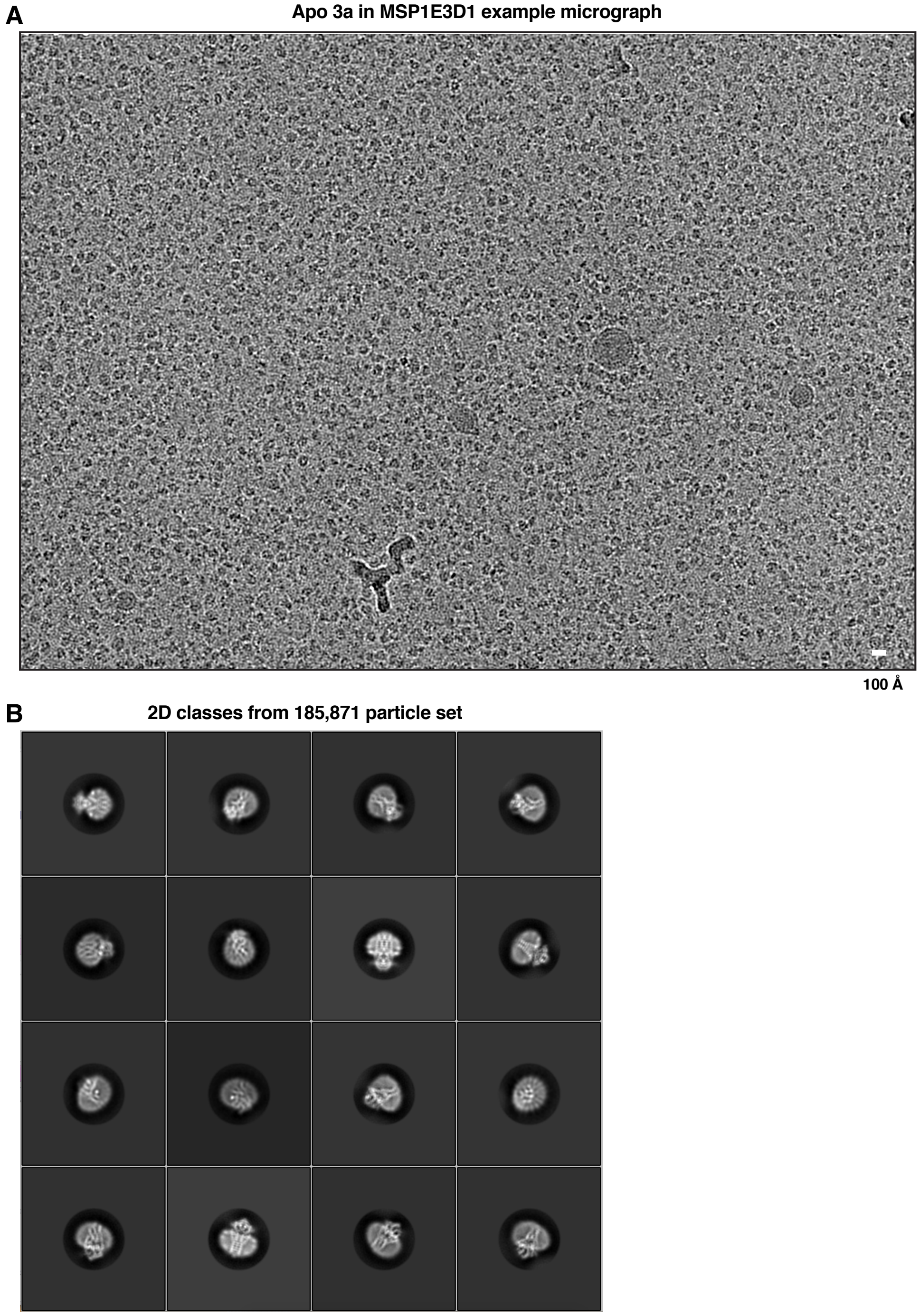

Figure S3 - Example micrographs and 2D class averages of dimeric apo 3a in MSP1E3D1 lipid nanodiscs
(A) Representative micrograph and (B) 2D class averages of dimeric apo 3a in MSP1E3D1 lipid nanodiscs.

bioRxiv preprint doi: https://doi.org/10.1101/2020.06.17.156554; this version posted January 26, 2021. The copyright holder for this preprint (which was not certified by peer review) is the author/funder, who has granted bioRxiv a license to display the preprint in perpetuity. It is made available under aCC-BY 4.0 International license.

Figure S4

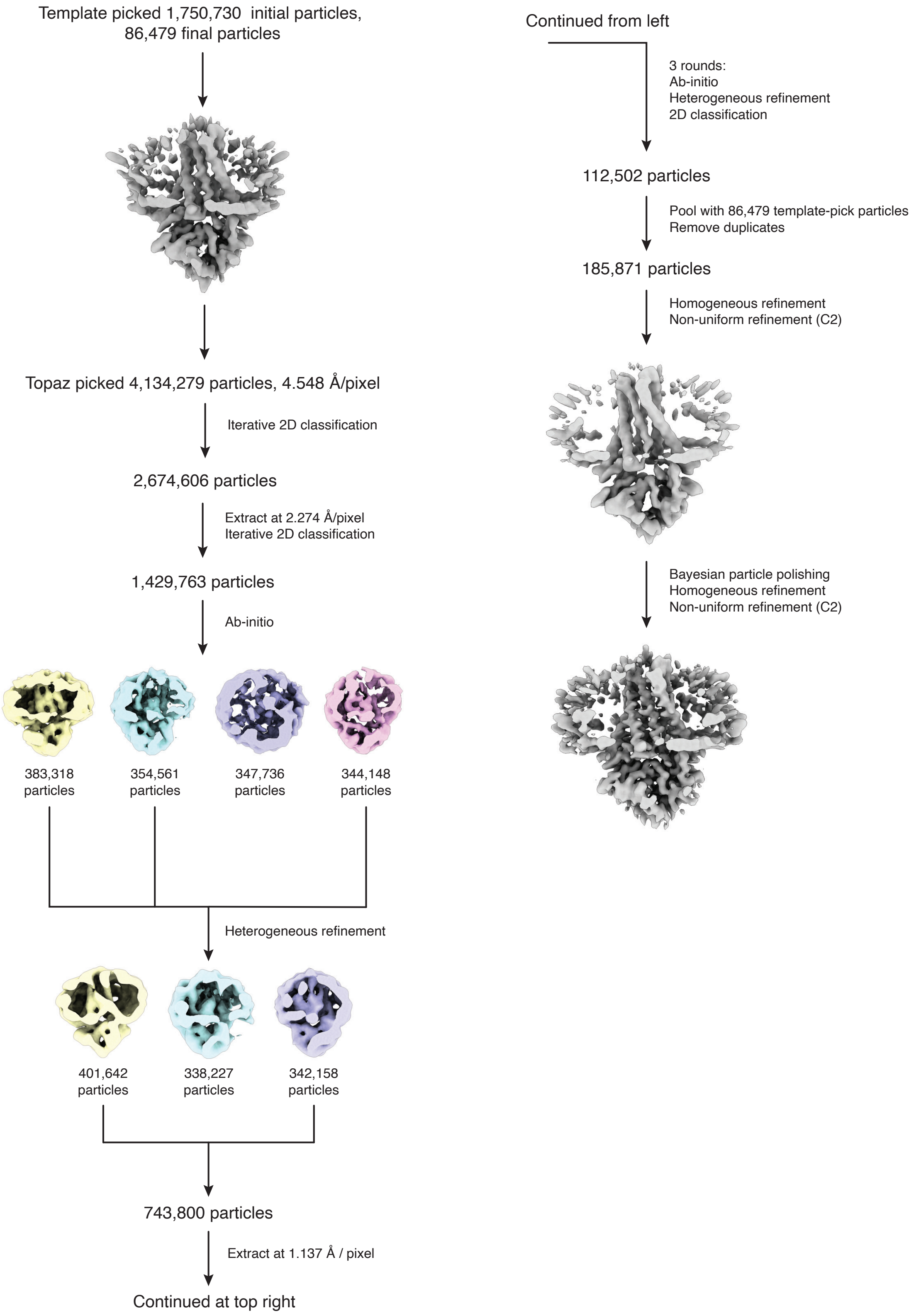

Figure S4 - Cryo-EM processing pipeline for dimeric apo 3a in MSP1E3D1 lipid nanodiscs. Overview of Cryo-EM data processing pipeline in cryoSPARC and Relion. See Methods for details.

bioRxiv preprint doi: https://doi.org/10.1101/2020.06.17.156554; this version posted January 26, 2021. The copyright holder for this preprint (which was not certified by peer review) is the author/funder, who has granted bioRxiv a license to display the preprint in perpetuity. It is made available under aCC-BY 4.0 International license.

Figure S5

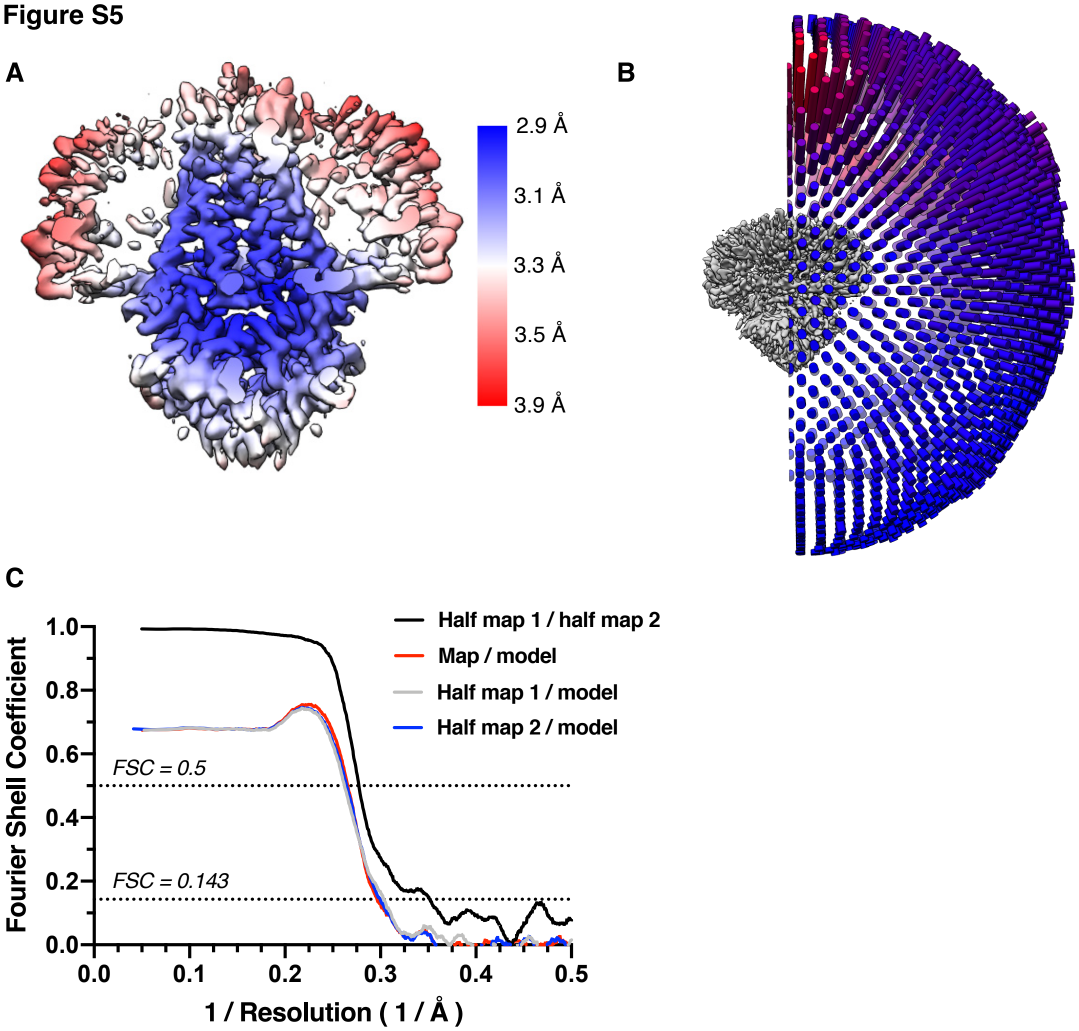

Figure S5 - Cryo-EM validation for dimeric apo 3a in MSP1E3D1 lipid nanodiscs.
(A) Local resolution estimated in Relion colored as indicated on the final map. (B) Angular distribution of particles used in final refinement with final map for reference. (C) Fourier Shell Correlation (FSC) relationships (unmasked) between (black) the two unfiltered half-maps from refinement and used for calculating overall resolution at 0.143, (red) the final map and model, (gray) half-map one and model, and (blue) half-map and model.

bioRxiv preprint doi: https://doi.org/10.1101/2020.06.17.156554; this version posted January 26, 2021. The copyright holder for this preprint (which was not certified by peer review) is the author/funder, who has granted bioRxiv a license to display the preprint in perpetuity. It is made available under aCC-BY 4.0 International license.

Figure S6

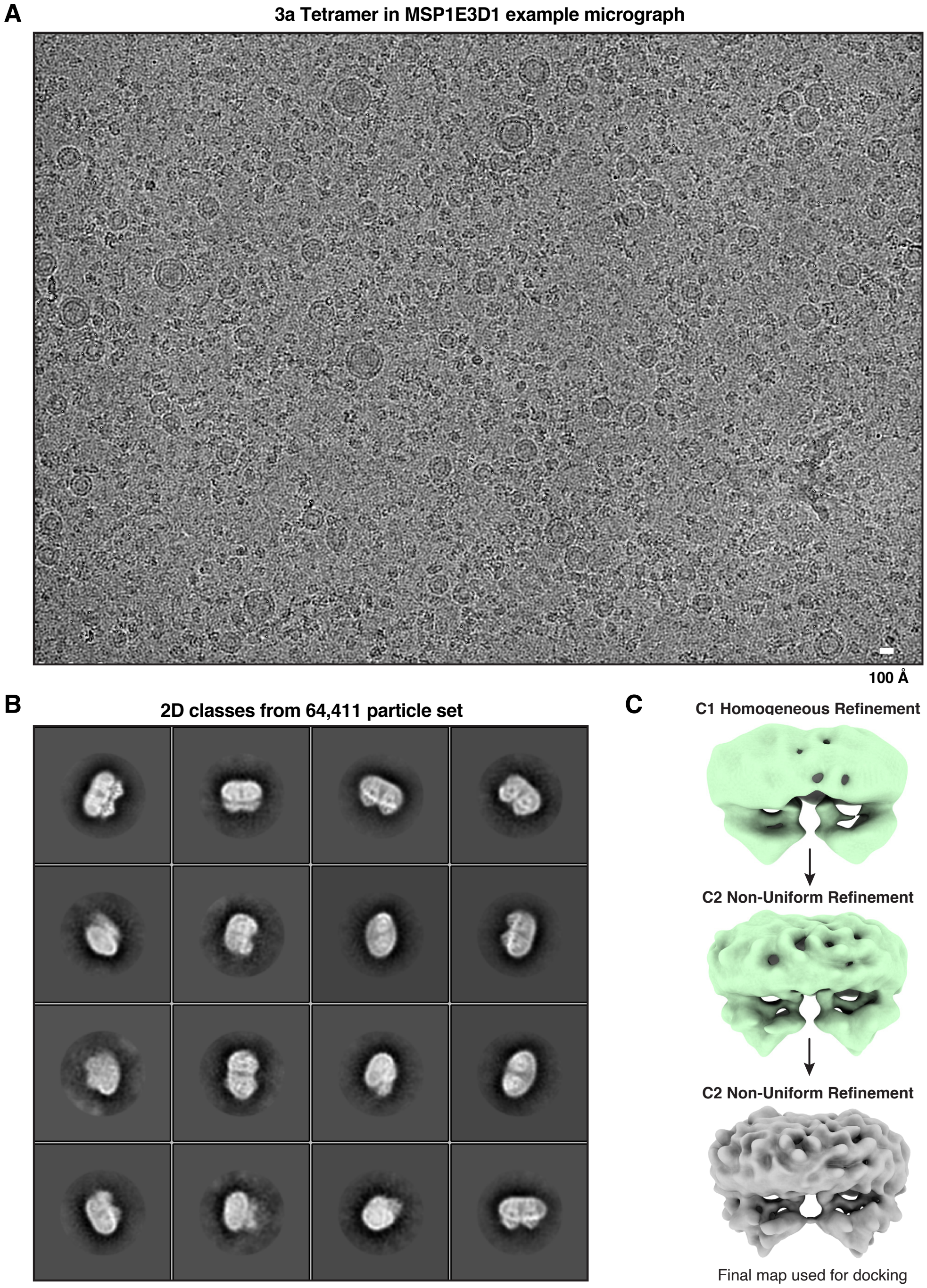

Figure S6 - Example micrographs, 2D class averages, and cryo-EM processing pipeline of tetrameric apo 3a in MSP1E3D1 lipid nanodiscs.

(A) Representative micrograph and (B) 2D class averages of tetrameric apo 3a in MSP1E3D1 lipid nanodiscs. (C) Map overview pipeline for final steps of processing (Also see Methods).

bioRxiv preprint doi: https://doi.org/10.1101/2020.06.17.156554; this version posted January 26, 2021. The copyright holder for this preprint (which was not certified by peer review) is the author/funder, who has granted bioRxiv a license to display the preprint in perpetuity. It is made available under aCC-BY 4.0 International license.

Figure S7

A **3a in MSP1E3D1 from +Emodin example micrograph**

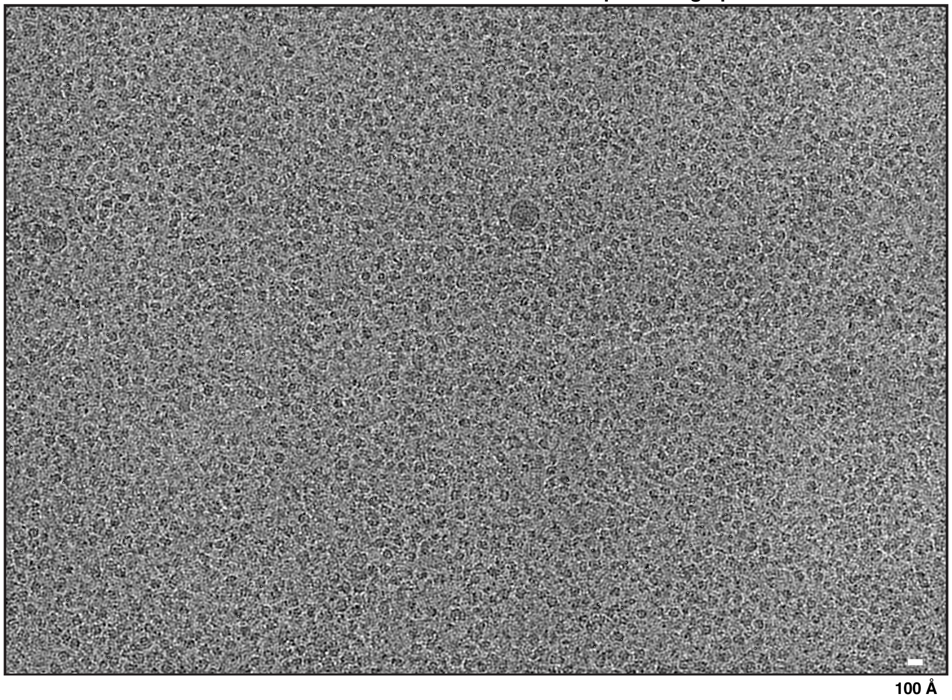

B **2D classes from final 51,908 particle set**

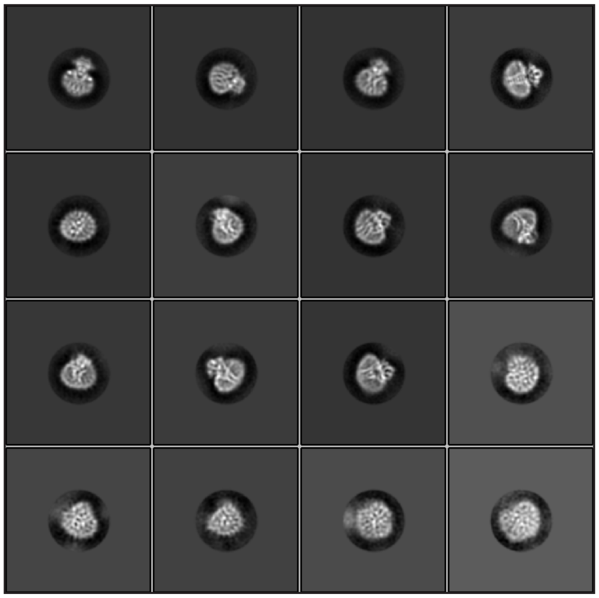

Figure S7 - Example micrographs and 2D class averages of dimeric 3a in MSP1E3D1 lipid nanodiscs with emodin added
(A) Representative micrograph and (B) 2D class averages of dimeric 3a in MSP1E3D1 lipid nanodiscs with emodin added.

bioRxiv preprint doi: https://doi.org/10.1101/2020.06.17.156554; this version posted January 26, 2021. The copyright holder for this preprint (which was not certified by peer review) is the author/funder, who has granted bioRxiv a license to display the preprint in perpetuity. It is made available under aCC-BY 4.0 International license.

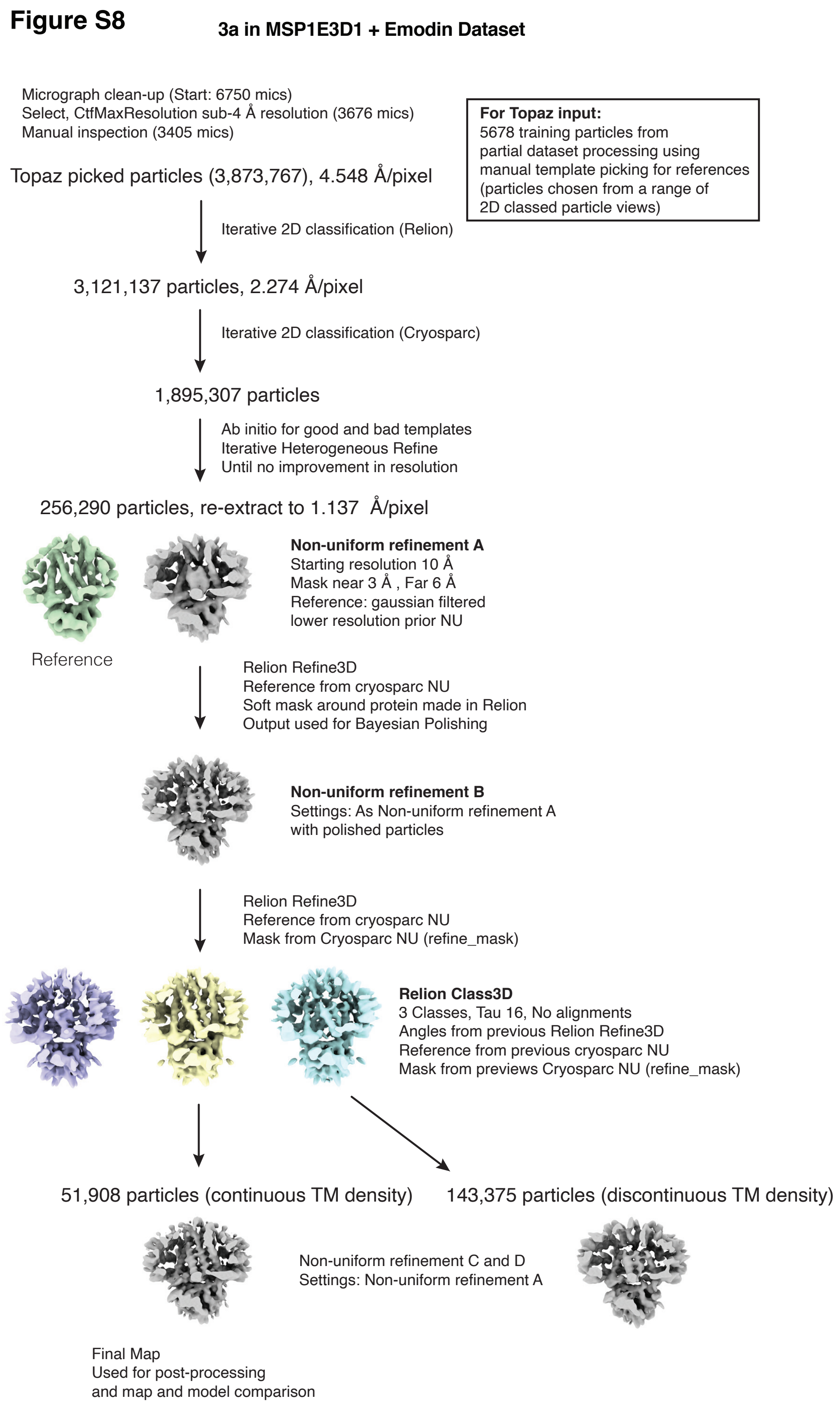

Figure S8 - Cryo-EM processing pipeline for dimeric 3a in MSP1E3D1 lipid nanodiscs with emodin added

Overview of Cryo-EM data processing pipeline in cryoSPARC and Relion. See Methods for details.

bioRxiv preprint doi: https://doi.org/10.1101/2020.06.17.156554; this version posted January 26, 2021. The copyright holder for this preprint (which was not certified by peer review) is the author/funder, who has granted bioRxiv a license to display the preprint in perpetuity. It is made available under aCC-BY 4.0 International license.

Figure S9

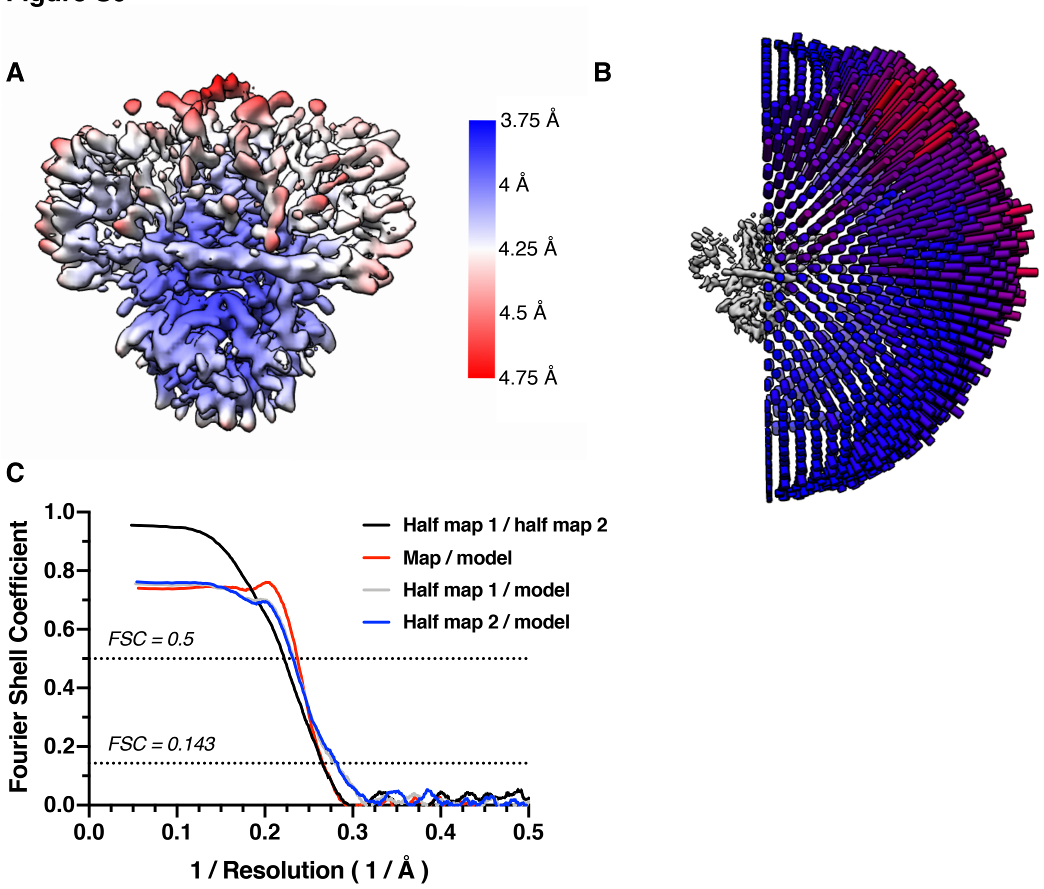

Figure S9 - Cryo-EM validation for dimeric 3a in MSP1E3D1 lipid nanodiscs with emodin added
(A) Local resolution estimated in Relion colored as indicated on the final map. (B) Angular distribution of particles used in final refinement with final map for reference. (C) Fourier Shell Correlation (FSC) relationships (masked) between (black) the two unfiltered half-maps from refinement and used for calculating overall resolution at 0.143, (red) the final map and model, (gray) half-map one and model, and (blue) half-map and model.

bioRxiv preprint doi: https://doi.org/10.1101/2020.06.17.156554; this version posted January 26, 2021. The copyright holder for this preprint (which was not certified by peer review) is the author/funder, who has granted bioRxiv a license to display the preprint in perpetuity. It is made available under aCC-BY 4.0 International license.

Figure S10

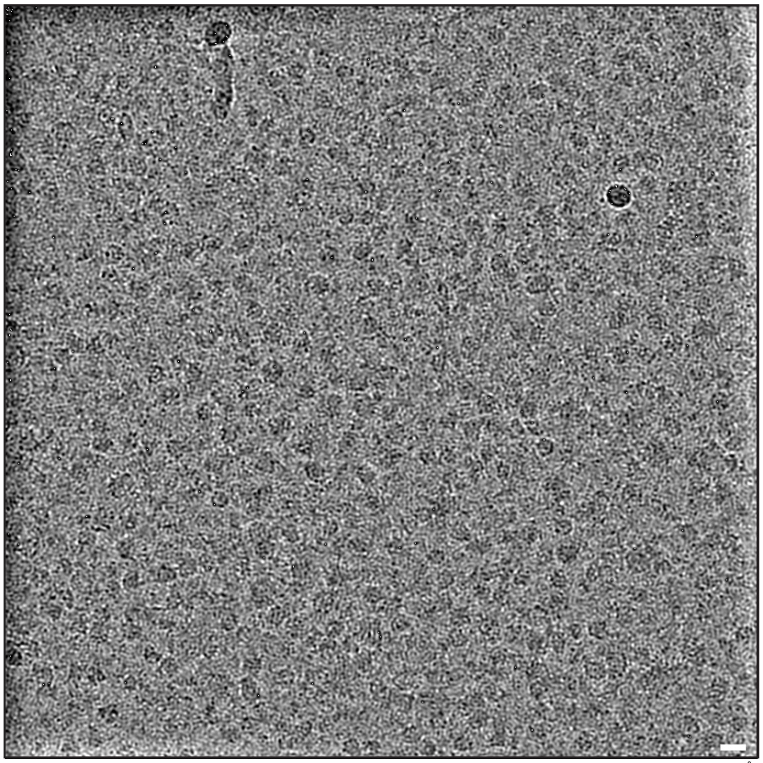

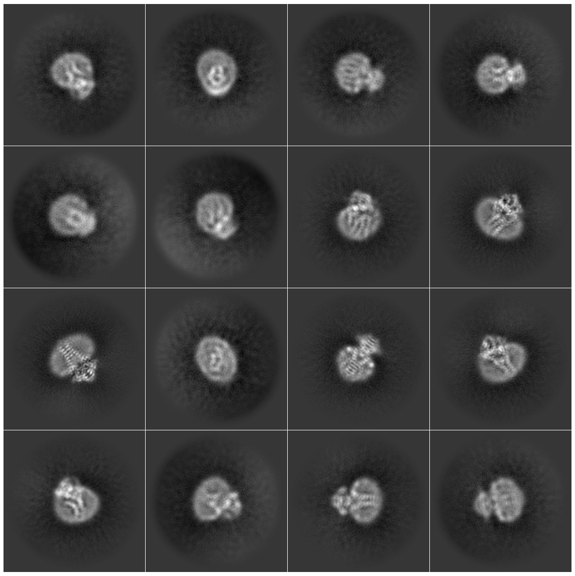

Figure S10 - Example micrographs and 2D class averages of dimeric apo 3a in MSP1E3D1 lipid nanodisc collected on the Krios with CFEG, Selectris, and Falcon 4
(A) Representative micrograph and (B) 2D class averages of dimeric apo 3a in MSP1E3D1 lipid nanodiscs from cryoSPARC.

bioRxiv preprint doi: https://doi.org/10.1101/2020.06.17.156554; this version posted January 26, 2021. The copyright holder for this preprint (which was not certified by peer review) is the author/funder, who has granted bioRxiv a license to display the preprint in perpetuity. It is made available under aCC-BY 4.0 International license.

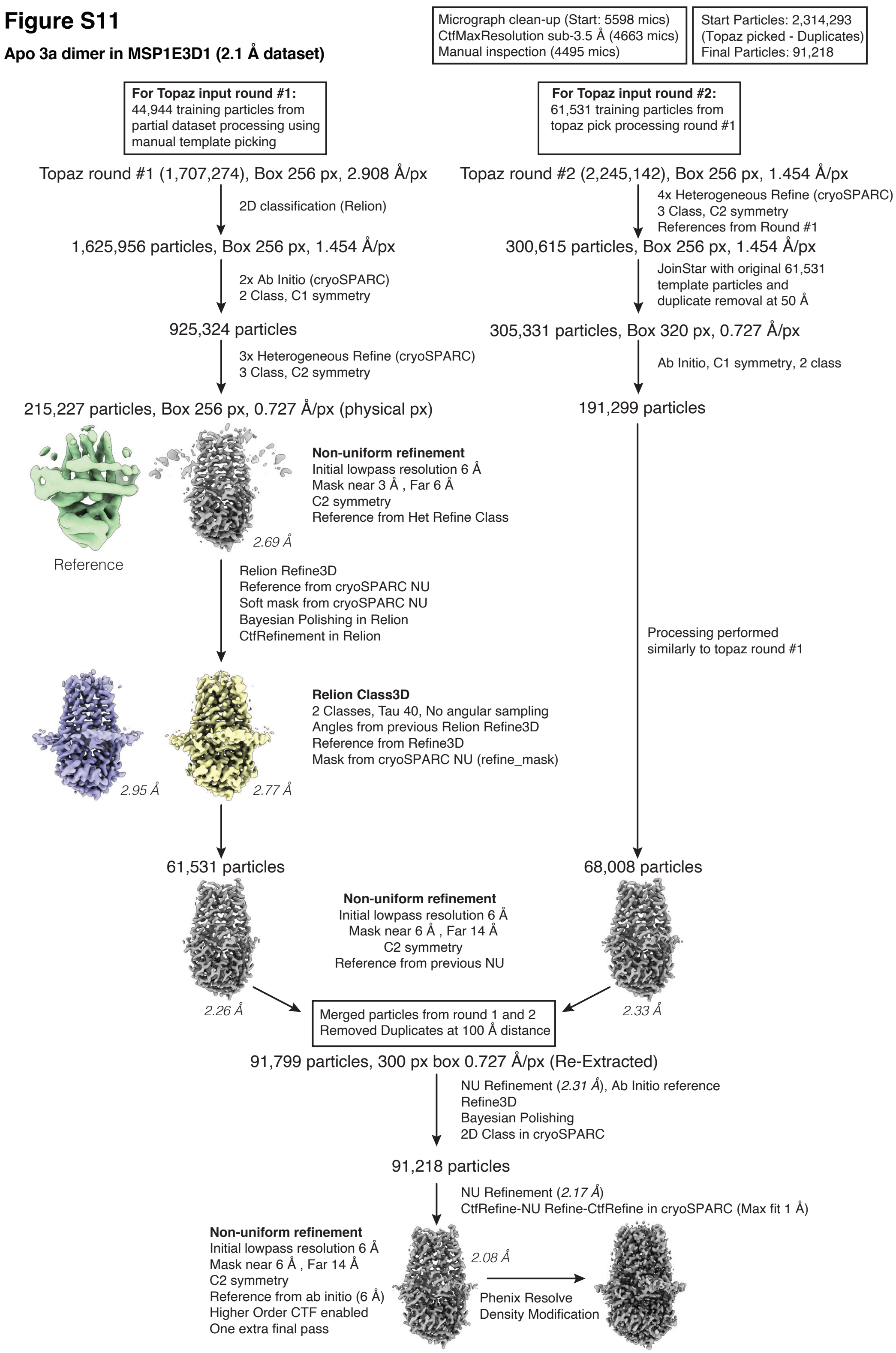

Figure S11 - Cryo-EM processing pipeline for dimeric apo 3a in MSP1E3D1 lipid nanodiscs. Overview of Cryo-EM data processing pipeline in cryoSPARC and Relion. See Methods for details.

bioRxiv preprint doi: https://doi.org/10.1101/2020.06.17.156554; this version posted January 26, 2021. The copyright holder for this preprint (which was not certified by peer review) is the author/funder, who has granted bioRxiv a license to display the preprint in perpetuity. It is made available under aCC-BY 4.0 International license.

Figure S12

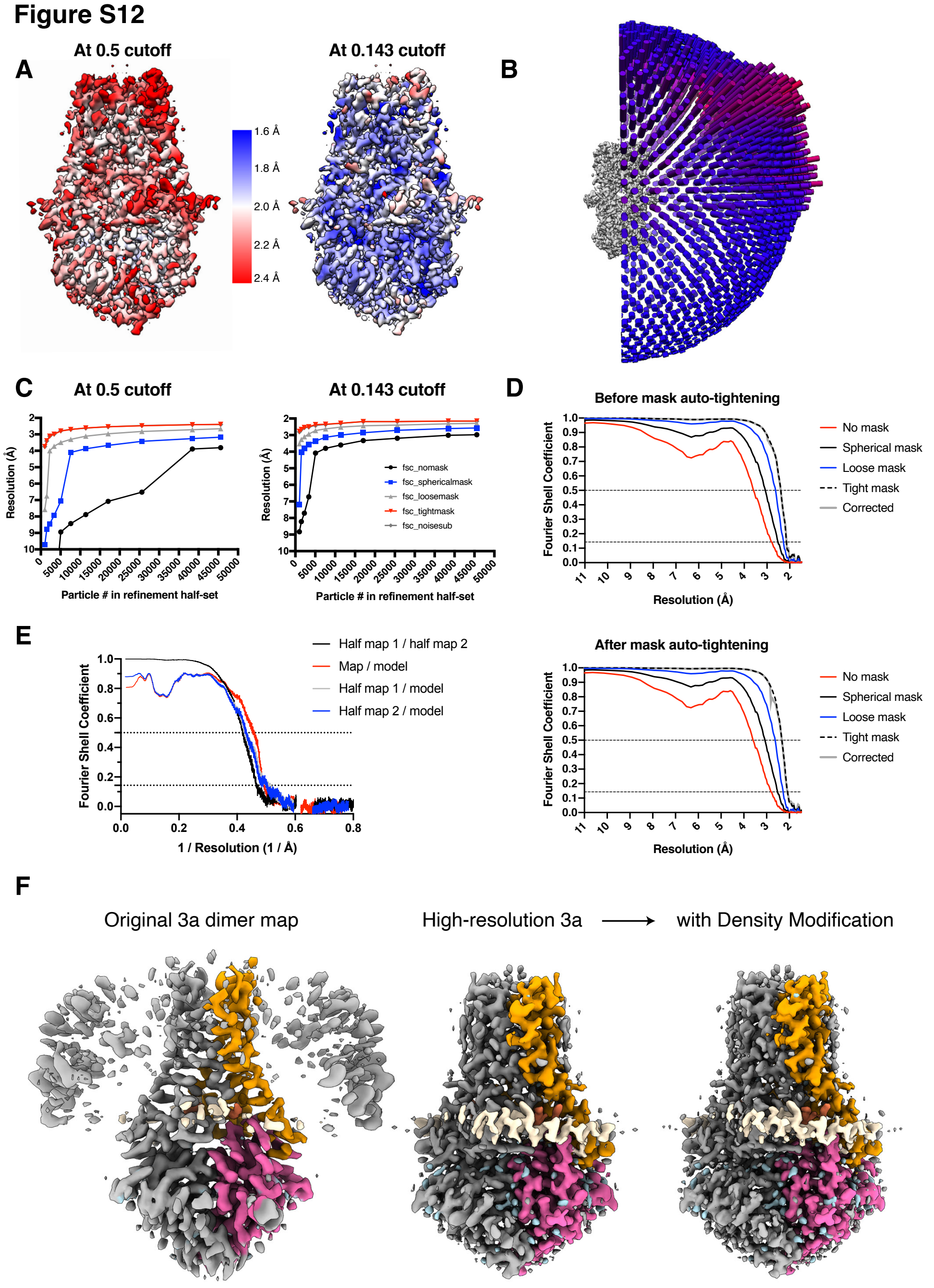

Figure S12 - Cryo-EM validation for dimeric apo 3a in MSP1E3D1 lipid nanodiscs.
(A) Local resolution estimated in cryoSPARC at the indicated FSC thresholds colored as indicated on the density modified map. (B) Angular distribution of particles used in final refinement with map for reference. (C) ResLog analysis conducted in cryoSPARC at the indicated FSC thresholds. (D) Fourier Shell Correlation as calculated in cryoSPARC before (top) and after (bottom) mask auto-tightening in the final round of refinement. (E) Fourier Shell Correlation (FSC) relationships (masked) calculated in Phenix between (black) the two unfiltered half-maps from refinement and used for calculating overall resolution at 0.143, (red) the final map and model, (gray) half-map one and model, and (blue) half-map and model. (F) Comparison of the original (2.9 Å) 3a map left to the high-resolution (2.1 Å) map before (middle) and after (right) Phenix density modification.

bioRxiv preprint doi: https://doi.org/10.1101/2020.06.17.156554; this version posted January 26, 2021. The copyright holder for this preprint (which was not certified by peer review) is the author/funder, who has granted bioRxiv a license to display the preprint in perpetuity. It is made available under aCC-BY 4.0 International license.

Figure S13

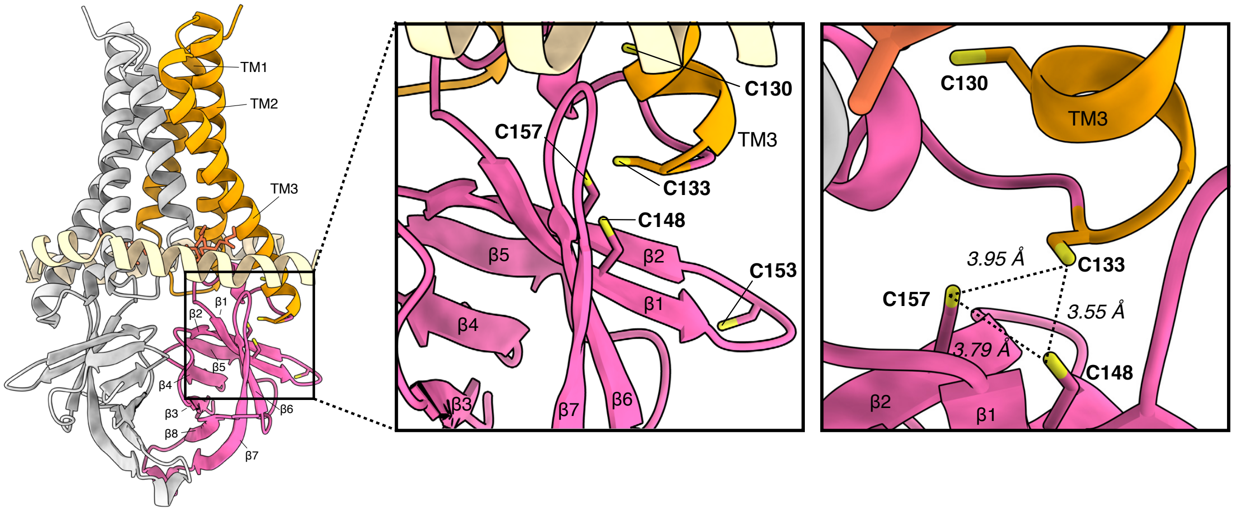

Figure S13 – A cysteine rich pocket in 3a
Full model (left) with boxed region zoom-in (middle) and an alternate view (right) to show the cysteine-rich region of 3a. Distances (dotted lines) between the reduced cysteines are displayed.

bioRxiv preprint doi: https://doi.org/10.1101/2020.06.17.156554; this version posted January 26, 2021. The copyright holder for this preprint (which was not certified by peer review) is the author/funder, who has granted bioRxiv a license to display the preprint in perpetuity. It is made available under aCC-BY 4.0 International license.

Figure S14

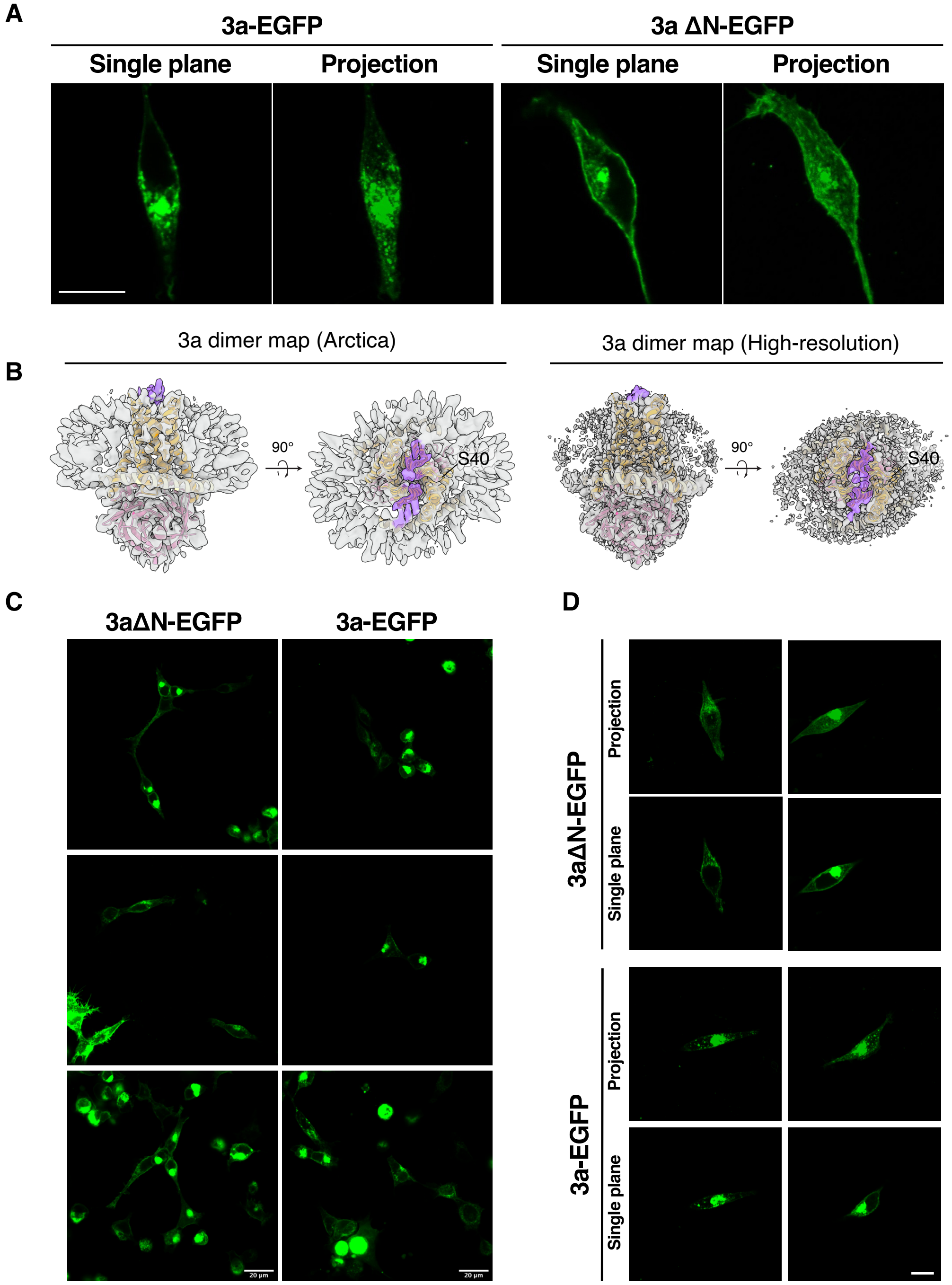

Figure S14 – Localization of 3a-EGFP and 3aΔN-EGFP expressed in HEK cells
(A) 3a-GFP fluorescence localization in HEK293 cells transfected with 3a-EGFP or 3aΔN-EGFP. Single plane and brightest-point projection are displayed for each. Scale bar, 10 μm. (B) Side view and view from the extracellular/lumenal space for dimeric 3a cryo-EM density (gray) from the original (left) and high-resolution (right) maps with unmodeled extended density above the mouth of the pore that may correspond to the N-terminal regions colored in purple. A 3a dimer model is drawn orange (TMD) and pink (CD) inside the density. The position of the final modeled N-terminal residue (S40) is indicated. (C) 3a-EGFP or 3aΔN-EGFP field of view with multiple cells imaged using a 20X objective. Scale bar, 20 μm. (D) Additional images of cells imaged with the 63X objective with both single plane and brightest-point projections displayed. Scale bar, 10 μm.

bioRxiv preprint doi: https://doi.org/10.1101/2020.06.17.156554; this version posted January 26, 2021. The copyright holder for this preprint (which was not certified by peer review) is the author/funder, who has granted bioRxiv a license to display the preprint in perpetuity. It is made available under aCC-BY 4.0 International license.

Figure S15

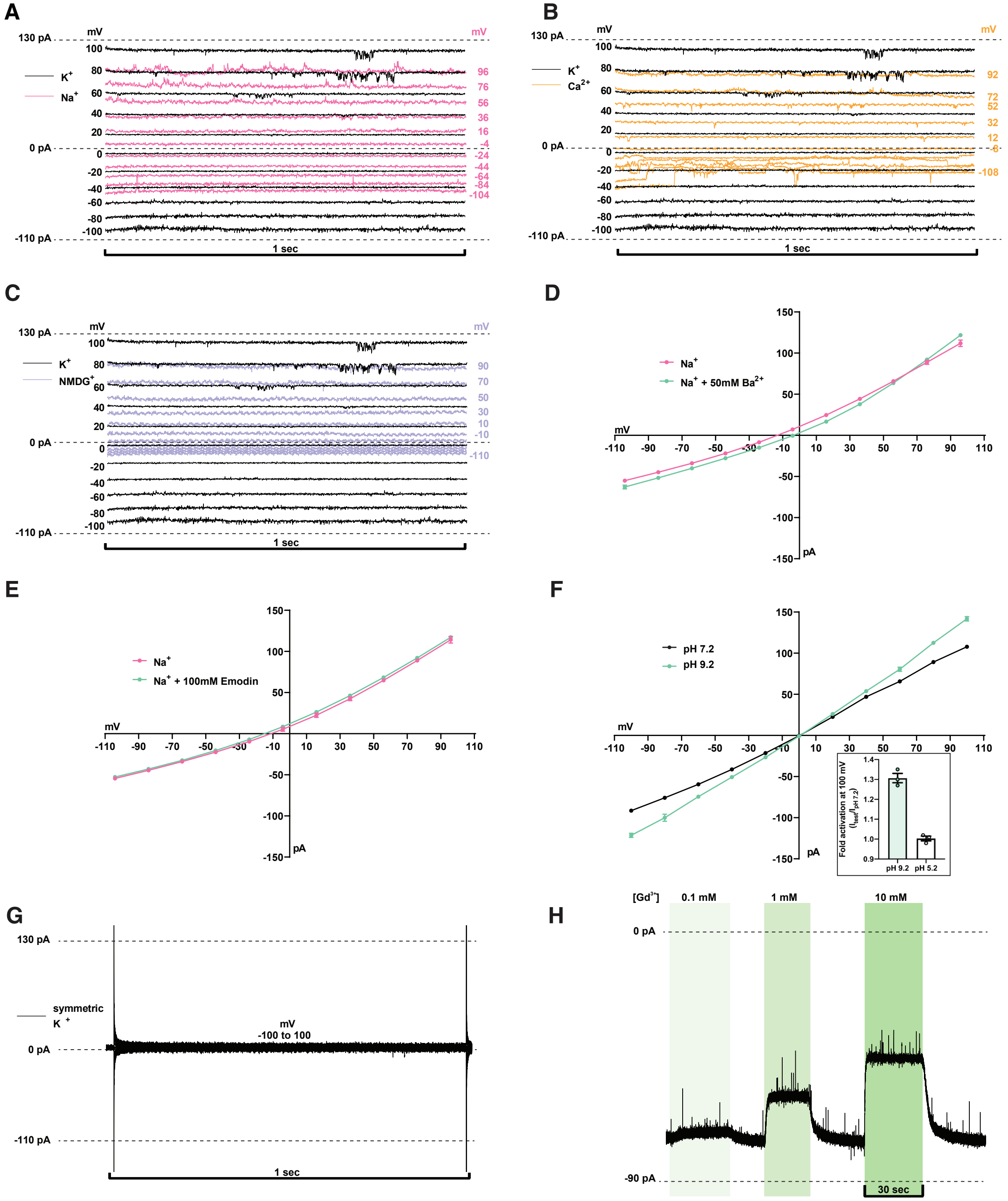

Figure S15 - Patch recordings from 3a-proteoliposomes

(A-C,G) Representative current recordings from 3a-proteoliposome. Currents were recorded with the following protocol: V_{hold} = 0 mV, V_{test} = -100 to 100 mV, ΔV = 20 mV, t_{test}=1 sec. Voltages indicated were corrected after recording for liquid junction potential. (A) 150 mM K^+ (black) or 150 mM Na^+ (pink) bath solution. (B) 150 mM K^+ (black) or 75 mM Ca^{2+} (orange) bath solution. (C) K^+ (black) or 150 mM $NMDG^+$ (blue) bath solution. (D) Ba^{2+} does not block 3a currents. Current-voltage relationship plotted from a recording in 150 mM Na^+ (pink) or 150 mM Na^+ with 50 mM Ba^{2+} (green) bath solution. (E) Emodin does

bioRxiv preprint doi: https://doi.org/10.1101/2020.06.17.156554; this version posted January 26, 2021. The copyright holder for this preprint (which was not certified by peer review) is the author/funder, who has granted bioRxiv a license to display the preprint in perpetuity. It is made available under aCC-BY 4.0 International license.

not block 3a currents. Current-voltage relationship plotted from a recording in 150 mM Na^+ (pink) or 150 mM Na^+ with 100 μM emodin (green) bath solution. (F) pH sensitivity of 3a. Current-voltage relationship plotted from a recording in 150 mM K^+ pH 7.2 (black) and 150 mM K^+ pH 9.2 (green) bath solution. (inset) Fold activation at +100 mV at pH 9.2 or pH 5.2 compared to pH 7.2. (G) Representative current recordings from mock reconstituted (empty) liposome patch. (H) Gap-free current recording in symmetric 150 mM KCl held at −80 mV during bath solution exchange and washout of varying Gd^{3+} concentrations represented by vertical green bars.

bioRxiv preprint doi: https://doi.org/10.1101/2020.06.17.156554; this version posted January 26, 2021. The copyright holder for this preprint (which was not certified by peer review) is the author/funder, who has granted bioRxiv a license to display the preprint in perpetuity. It is made available under aCC-BY 4.0 International license.

Figure S16

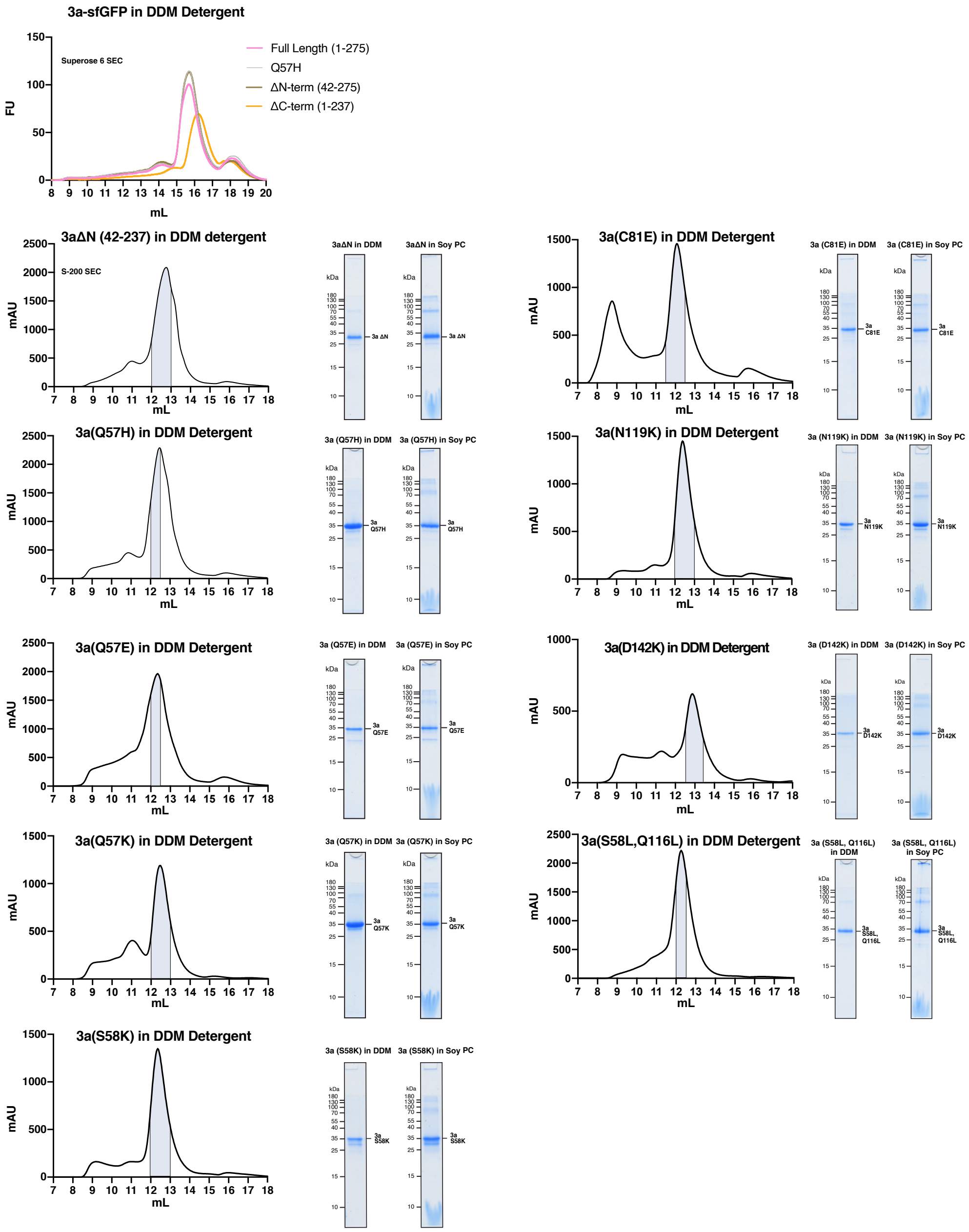

Figure S16 – Purification and liposome reconstitution of 3a mutants and truncation

Top: GFP fluorescence chromatogram (FSEC) of 3a, as well as indicated mutants and truncations, expressed in SF9 cells and extracted in DDM detergent. Samples were run on a Superose 6 column. For all other panels: Size exclusion chromatogram from a s200 column of indicated 3a constructs expressed in insect cells and extracted and purified in DDM (left), coomassie-stained SDS-PAGE of pooled dimeric 3a construct -containing fractions (center), and of 3a following reconstitution into PC lipids (right).

bioRxiv preprint doi: https://doi.org/10.1101/2020.06.17.156554; this version posted January 26, 2021. The copyright holder for this preprint (which was not certified by peer review) is the author/funder, who has granted bioRxiv a license to display the preprint in perpetuity. It is made available under aCC-BY 4.0 International license.

Figure S17

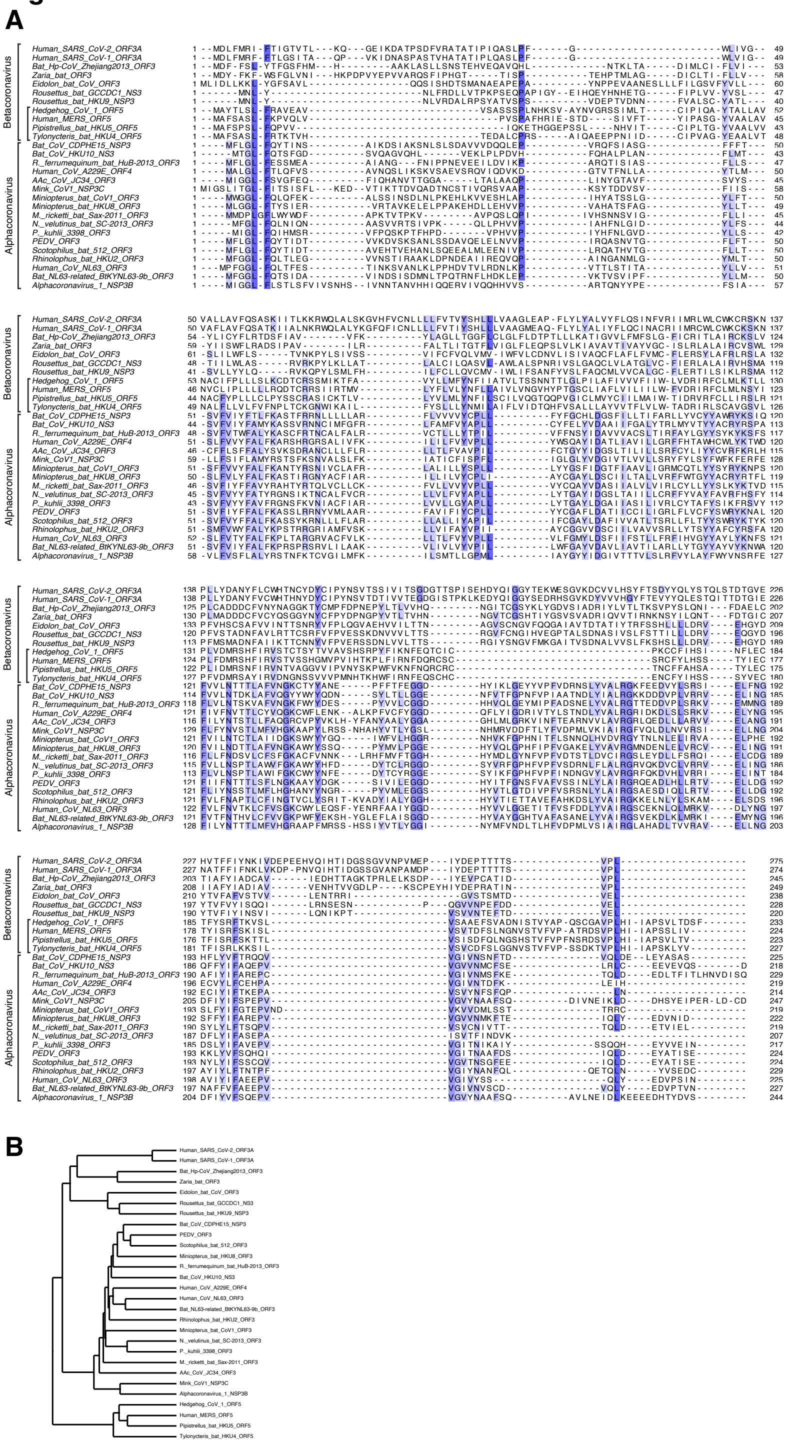

Figure S17 - Sequence alignment of 3a-like proteins across *Coronaviridae*
(A) Alignment of twenty-eight 3a-like protein sequences colored by conservation in a ramp from white (not conserved) to dark blue (highly conserved). Accession numbers are listed in Table 2. Sequences were selected from representative species from each Coronavirus subgenus. *Alphacoronavirus* and Betacoronavirus clades are indicated. Within Betacoronavirus the subgenus *Merbecovirus* is also indicated with a bar. (B) Neighbor-joining tree calculated from the alignment in (A).

bioRxiv preprint doi: https://doi.org/10.1101/2020.06.17.156554; this version posted January 26, 2021. The copyright holder for this preprint (which was not certified by peer review) is the author/funder, who has granted bioRxiv a license to display the preprint in perpetuity. It is made available under aCC-BY 4.0 International license.

**Data collection**	**Dimeric apo 3a (2.9 Å)**	**Dimeric 3a with emodin**	**Tetrameric apo 3a**	**Dimeric apo 3a (2.1 Å)**
PDB	6XDC	n/a	n/a	7KJR
EMDB	22136	22139	22138	22898
EMPIAR	10439	10440	10441	10612
Total movies #	6309	6750	7092	5598
Selected movies #	2595	3405	4324	4495
Magnification	36,000 x	36,000 x	36,000 x	165,000 x
Voltage (KV)	200	200	200	300
Electron exposure ($e^-/Å^2$)	50.325 or 53.72 (1-2007) (2008-6309)	47.21	49.95	50
Frame #	50	50	50	1449 or 1379 (EER)
Defocus range (um)	-0.6 to -2.0	-0.6 to -2.0	-0.6 to -2.0	-0.5 to -1.1
Super resolution pixel size ($Å^2$)	0.5685	0.5685	0.5685	0.3685
Binned pixel size ($Å^2$)	1.137	1.137	1.137	0.727
**Processing**				
Initial particle images (no.)	4,134,279	3,873,767	1,282,913	2,314,293
Final particle images (no.)	185,871	51,908	64,410	91,218
Map resolution Masked (Å, FSC = 0.143)	2.9	3.69	6.5	2.08
Symmetry imposed	C2	C2	C2	C2
**Refinement**				
Model resolution (Å, FSC = 0.143 / FSC = 0.5)	3.2/3.6			2.0 / 2.2
Map-sharpening B factor ($Å^2$)	-111.2			-43.5
Composition				
Number of atoms	3150			3840
Number of protein residues	386			448
Number of ligands				2
Number of waters				122
R.m.s. deviations				
Bond lengths (Å)	0.006			0.005
Bond angles (Å)	0.785			0.729
Validation				
MolProbity score	1.55			1.42
Clashscore	4.63			7.60
Ramachandran plot				
Favored (%)	96.03			98.17
Allowed (%)	3.97			1.13
Disallowed (%)	0			0
Rotamer outliers (%)	1.15			0.25
Mean B factor ($Å^2$)				
Protein	108.61			23.30
Ligand				51.72
Water				22.11

Table S1 - Cryo-EM data collection, processing, refinement, and modeling data

bioRxiv preprint doi: https://doi.org/10.1101/2020.06.17.156554; this version posted January 26, 2021. The copyright holder for this preprint (which was not certified by peer review) is the author/funder, who has granted bioRxiv a license to display the preprint in perpetuity. It is made available under aCC-BY 4.0 International license.

Proposed host origin		Subgenus / Species	Annotated ORF name	Accession	Phyre predicted amino acid range	Phyre confidence score
Bat	Alphacoronavirus	**Colacovirus**				
		Bat coronavirus CDPHE15 (1384461)	NS3	YP_008439203.1	69 - 222	98%
		**Decacovirus**				
		Bat coronavirus HKU10 (1244203)	NS3	AFU92105.1	69 - 221	96%
		R. ferrumequinum HuB-2013 (2501926)	ORF3	YP_009199791.1	69 - 220	97%
		**Duvinacovirus**				
		Human coronavirus 229E (11137)	ORF4	ARU07602.1	69 - 221	95%
		**Luchacovirus**				
		Coronavirus AcCoV-JC34 (1964806)	ORF3	YP_009380522.1	69 - 220	96%
		**Minacovirus**				
		Mink coronavirus 1 (766791)	NS3	YP_009019183.1	68-221	96%
		**Minunacovirus**				
		Miniopterus bat coronavirus 1 (694000)	ORF3	ACA52165.1	69 - 221	96%
		Miniopterus bat coronavirus HKU8 (694001)	ORF3	AIA62228.1	69 - 221	96%
		**Myotacovirus**				
		Myotis ricketti alphacoronavirus Sax-2011 (2501927)	ORF3	AIA62247.1	78-211	97%
		**Nyctacovirus**				
		Nyctalus velutinus alphacoronavirus SC-2013 (2501928)	ORF3	YP_009201731.1	69 - 220	97%
		Pipistrellus kuhlii coronavirus 3398 (2492658)	ORF3	YP_009755891.1	69 - 220	97%
		**Pedacovirus**				
		Porcine epidemic diarrhea virus (28295)	ORF3	AWM99571.1	69 - 221	96%
		Scotophilus bat coronavirus 512 (693999)	ORF3	YP_001351685.1	69 - 220	97%
		**Rhinacovirus**				
		Rhinolophus bat coronavirus HKU2 (693998)	ORF3	ATN23890.1	69 - 220	97%
		**Setracovirus**				
		Human coronavirus NL63 (277944)	ORF3	AGT51388.1	69 - 221	97%
		NL63-related bat coronavirus BtKYNL63-9b (1920748)	ORF3	YP_009824968.1	69 - 220	96%
		**Tegacovirus**				
		Alphacoronavirus 1 (693997)	NSP3B	AEM55568.1	69 - 220	96%
	Betacoronavirus	**Sarbecovirus**				
		Human SARS-CoV-1 (694009)	ORF3A	P59632	42-237	100%
		Human SARS-CoV-2 (2697049)	ORF3A	YP_009724391.1	n/a	n/a
		**Hibecovirus**				
		Bat Hp-betacoronavirus Zhejiang2013 (1541205)	ORF3	YP_009072441.1	42-236	100%
		Zaria bat coronavirus (989337)	ORF3	ADY17912.1	101-233	100%
		**Nobecovirus**				
		Eidolon bat coronavirus C704 (983924)	ORF3	ADX59467.1	123-190	96%
		Rousettus bat coronavirus GCCDC1 (1892416)	NS3	YP_009273006.1	122-209	95%
		Rousettus bat coronavirus HKU9 (694006)	NS3	QJX58367.1	122-209	95%
		**Merbecovirus**				
		Human Middle East respiratory syndrome-related (1335626)	ORF5	QJX19961.1	119-146	88%
		Hedgehog coronavirus 1 (1965093)	ORF5	QCC20718.1	126-205	86%
		Pipistrellus bat coronavirus HKU5 (694008)	ORF5	AWH65914.1	126-182	92%
		Tylonycteris bat coronavirus HKU4 (694007)	ORF5	AWH65903.1	126-182	91%
Rodent		**Embecovirus**				
		Betacoronavirus 1 (694003)	none	none	none	none
		China Rattus coronavirus HKU24 (2501960)	none	none	none	none
		Human coronavirus HKU1 (290028)	none	none	none	none
		Murine coronavirus (694005)	none	none	none	none
		Myodes coronavirus 2JL14	none	none	none	none
Avian / Pig	Deltacoronavirus	**Andecovirus**				
		Wigeon coronavirus HKU20 (1159908)	none	none	none	none
		**Buldecovirus**				
		Bulbul coronavirus HKU11 (574549)	none	none	none	none
		Common moorhen coronavirus HKU21	none	none	none	none
		Coronavirus HKU15 (1965089)	none	none	none	none
		Munia coronavirus HKU13 (1297661)	none	none	none	none
		White-eye coronavirus HKU16 (1159907)	none	none	none	none
		**Herdecovirus**				
		Night heron coronavirus HKU19	none	none	none	none
	Gammacoronavirus	**Brangacovirus**				
		Goose coronavirus CB17	none	none	none	none
		**Cegacovirus**				
		Beluga whale coronavirus SW1 (694015)	none	none	none	none
		**Igacovirus**				
		Avian coronavirus (694014)	none	none	none	none
		Avian coronavirus 9203	none	none	none	none
		Duck coronavirus 2714 (300188)	none	none	none	none

Table S2 - 3a homologs across *Coronaviridae*
