# Supplementary material for: Cryo-EM structure of the SARS-CoV-2 3a ion channel in lipid nanodiscs
Source: bioRxiv. 2020 Jun 25:2020.06.17.156554. Originally published 2020 Jun 18. Preprint. [Version 2] doi: 10.1101/2020.06.17.156554 (PMC7310636; doi:10.1101/2020.06.17.156554)
Supplement: Supplementary file 1 [file NIHPP2020.06.17.156554-supplement-1.pdf]

bioRxiv preprint doi: https://doi.org/10.1101/2020.06.17.156554. this version posted June 25, 2020. The copyright holder for this preprint (which was not certified by peer review) is the author/funder. It is made available under a CC-BY 4.0 International license.

**Data collection**	**Dimeric apo 3a**	**Dimeric 3a with emodin added**	**Tetrameric apo 3a**
PDB	6XDC	n/a	n/a
EMDB	22136	22139	22138
EMPIAR	10439	10440	10441
Total movies #	6309	6750	7092
Selected movies #	2595	3405	4324
Magnification	36,000 x	36,000 x	36,000 x
Voltage (KV)	200	200	200
Electron exposure (e^-/Å^2)	50.325 or 53.72 (1-2007) (2008-6309)	47.21	49.95
Frame #	50	50	50
Defocus range (um)	-0.6 to -2.0	-0.6 to -2.0	-0.6 to -2.0
Super resolution pixel size (Å^2)	0.5685	0.5685	0.5685
Binned pixel size (Å^2)	1.137	1.137	1.137
**Processing**			
Initial particle images (no.)	4,134,279	3,873,767	1,282,913
Final particle images (no.)	185,871	51,908	64,410
Map resolution Masked (Å, FSC = 0.143)	2.9	3.69	6.5
Symmetry imposed	C2	C2	C2
**Refinement**			
Model resolution (Å, FSC = 0.143 / FSC = 0.5)	3.2/3.6		
Map-sharpening B factor (Å^2)	-111.2		
Composition			
Number of atoms	3150		
Number of protein residues	386		
R.m.s. deviations			
Bond lengths (Å)	0.006		
Bond angles (Å)	0.785		
Validation			
MolProbity score	1.55		
Clashscore	4.63		
Ramachandran plot			
Favored (%)	96.03		
Allowed (%)	3.97		
Disallowed (%)	0		
Rotamer outliers (%)	1.15		
Mean B factor (Å^2)			
Protein	108.61		

Table S1 - Cryo-EM data collection, processing, refinement, and modeling data

bioRxiv preprint doi: https://doi.org/10.1101/2020.06.17.156554. this version posted June 25, 2020. The copyright holder for this preprint (which was not certified by peer review) is the author/funder. It is made available under a CC-BY 4.0 International license.

Proposed host origin		Subgenus / Species	Annotated ORF name	Accession	Phyre predicted amino acid range	Phyre confidence score
Bat	Alphacoronavirus	Colacovirus				
		Bat coronavirus CDPHE15 (1384461)	NS3	YP_008439203.1	69 - 222	98%
		Decacovirus				
		Bat coronavirus HKU10 (1244203)	NS3	AFU92105.1	69 - 221	96%
		R. ferrumequinum HuB-2013 (2501926)	ORF3	YP_009199791.1	69 - 220	97%
		Duvinacovirus				
		Human coronavirus 229E (11137)	ORF4	ARU07602.1	69 - 221	95%
		Luchacovirus				
		Coronavirus AcCoV-JC34 (1964806)	ORF3	YP_009380522.1	69 - 220	96%
		Minacovirus				
		Mink coronavirus 1 (766791)	NS3	YP_009019183.1	68-221	96%
		Minunacovirus				
		Miniopterus bat coronavirus 1 (694000)	ORF3	ACA52165.1	69 - 221	96%
		Miniopterus bat coronavirus HKU8 (694001)	ORF3	AIA62228.1	69 - 221	96%
		Myotacovirus				
		Myotis ricketti alphacoronavirus Sax-2011 (2501927)	ORF3	AIA62247.1	78-211	97%
		Nyctacovirus				
		Nyctalus velutinus alphacoronavirus SC-2013 (2501928)	ORF3	YP_009201731.1	69 - 220	97%
		Pipistrellus kuhlii coronavirus 3398 (2492658)	ORF3	YP_009755891.1	69 - 220	97%
		Pedacovirus				
		Porcine epidemic diarrhea virus (28295)	ORF3	AWM99571.1	69 - 221	96%
		Scotophilus bat coronavirus 512 (693999)	ORF3	YP_001351685.1	69 - 220	97%
		Rhinacovirus				
		Rhinolophus bat coronavirus HKU2 (693998)	ORF3	ATN23890.1	69 - 220	97%
		Setracovirus				
		Human coronavirus NL63 (277944)	ORF3	AGT51388.1	69 - 221	97%
		NL63-related bat coronavirus BtKYNL63-9b (1920748)	ORF3	YP_009824968.1	69 - 220	96%
		Tegacovirus				
		Alphacoronavirus 1 (693997)	NSP3B	AEM55568.1	69 - 220	96%
	Betacoronavirus	Sarbecovirus				
		Human SARS-CoV-1 (694009)	ORF3A	P59632	42-237	100%
		Human SARS-CoV-2 (2697049)	ORF3A	YP_009724391.1	n/a	n/a
		Hibecovirus				
		Bat Hp-betacoronavirus Zhejiang2013 (1541205)	ORF3	YP_009072441.1	42-236	100%
		Zaria bat coronavirus (989337)	ORF3	ADY17912.1	101-233	100%
		Nobecovirus				
		Eidolon bat coronavirus C704 (983924)	ORF3	ADX59467.1	123-190	96%
		Rousettus bat coronavirus GCCDC1 (1892416)	NS3	YP_009273006.1	122-209	95%
		Rousettus bat coronavirus HKU9 (694006)	NS3	QJX58367.1	122-209	95%
		Merbecovirus				
		Human Middle East respiratory syndrome-related (1335626)	ORF5	QJX19961.1	119-146	88%
		Hedgehog coronavirus 1 (1965093)	ORF5	QCC20718.1	126-205	86%
		Pipistrellus bat coronavirus HKU5 (694008)	ORF5	AWH65914.1	126-182	92%
		Tylonycteris bat coronavirus HKU4 (694007)	ORF5	AWH65903.1	126-182	91%
Rodent		Embecovirus				
		Betacoronavirus 1 (694003)	none	none	none	none
		China Rattus coronavirus HKU24 (2501960)	none	none	none	none
		Human coronavirus HKU1 (290028)	none	none	none	none
		Murine coronavirus (694005)	none	none	none	none
		Myodes coronavirus 2JL14	none	none	none	none
Avian / Pig	Deltacoronavirus	Andecovirus				
		Wigeon coronavirus HKU20 (1159908)	none	none	none	none
		Buldecovirus				
		Bulbul coronavirus HKU11 (574549)	none	none	none	none
		Common moorhen coronavirus HKU21	none	none	none	none
		Coronavirus HKU15 (1965089)	none	none	none	none
		Munia coronavirus HKU13 (1297661)	none	none	none	none
		White-eye coronavirus HKU16 (1159907)	none	none	none	none
		Herdecovirus				
		Night heron coronavirus HKU19	none	none	none	none
	Gammacoronavirus	Brangacovirus				
		Goose coronavirus CB17	none	none	none	none
		Cegacovirus				
		Beluga whale coronavirus SW1 (694015)	none	none	none	none
		Igacovirus				
		Avian coronavirus (694014)	none	none	none	none
		Avian coronavirus 9203	none	none	none	none
		Duck coronavirus 2714 (300188)	none	none	none	none

Table S2 - 3a homologs across *Coronaviridae*

bioRxiv preprint doi: https://doi.org/10.1101/2020.06.17.156554. this version posted June 25, 2020. The copyright holder for this preprint (which was not certified by peer review) is the author/funder. It is made available under a CC-BY 4.0 International license.

A

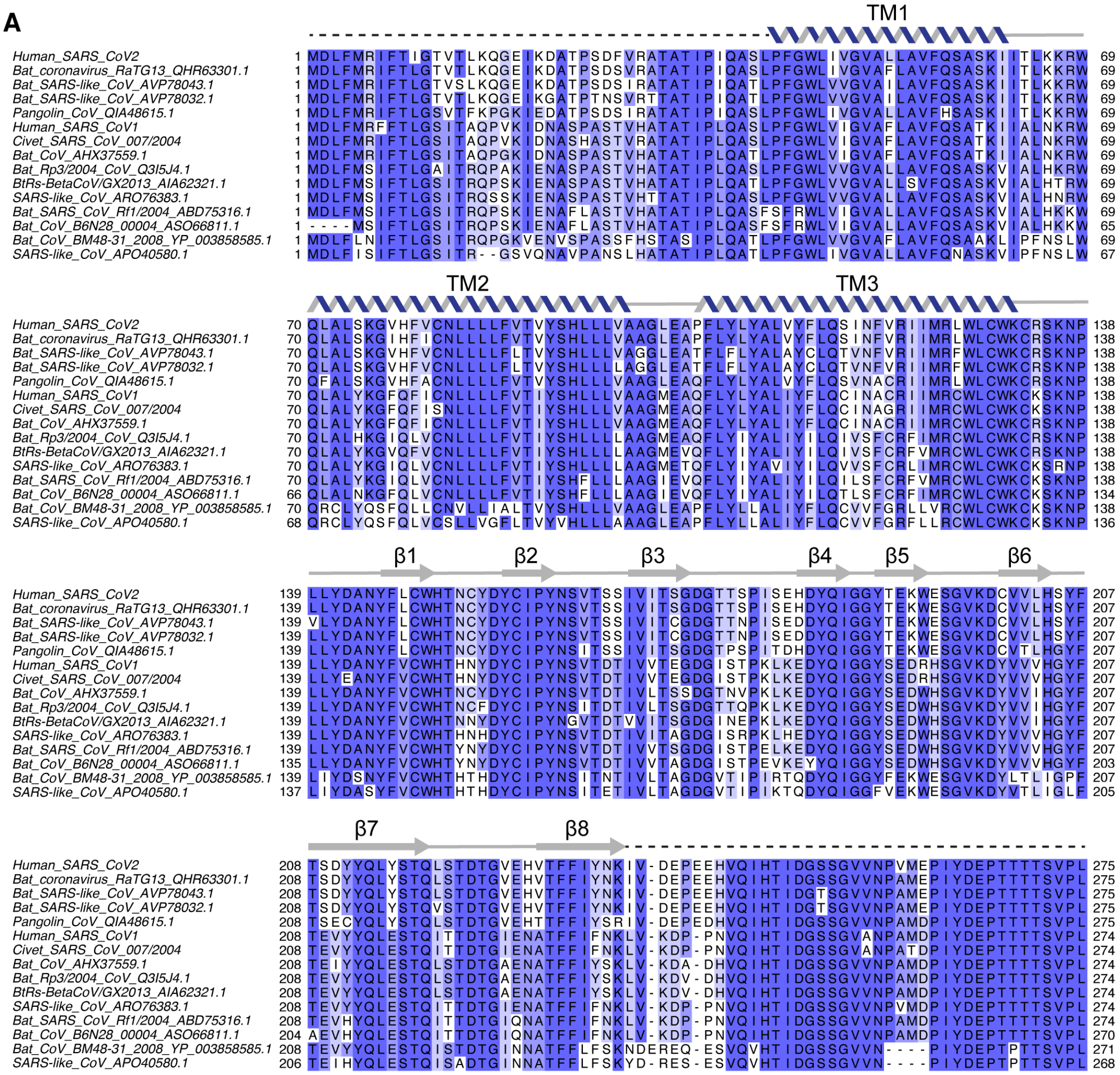

B

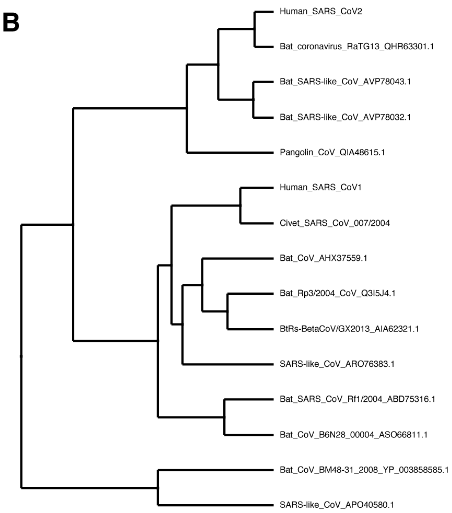

Figure S1 – Sequence alignment of 3a channels from *Betacoronavirus Sarbecovirus* (A) Alignment of fifteen 3a protein sequences colored by conservation in a ramp from white (not conserved) to dark blue (highly conserved). Accession numbers are indicated. Sequences were selected to maximize diversity among annotated *Sarbecovirus* 3a proteins. Secondary structure for SARS-CoV-2 is drawn above the sequence with unmodeled sequence drawn as dashed lines. (B) Neighbor-joining tree calculated from the alignment in (A).

bioRxiv preprint doi: https://doi.org/10.1101/2020.06.17.156554. this version posted June 25, 2020. The copyright holder for this preprint (which was not certified by peer review) is the author/funder. It is made available under a CC-BY 4.0 International license.

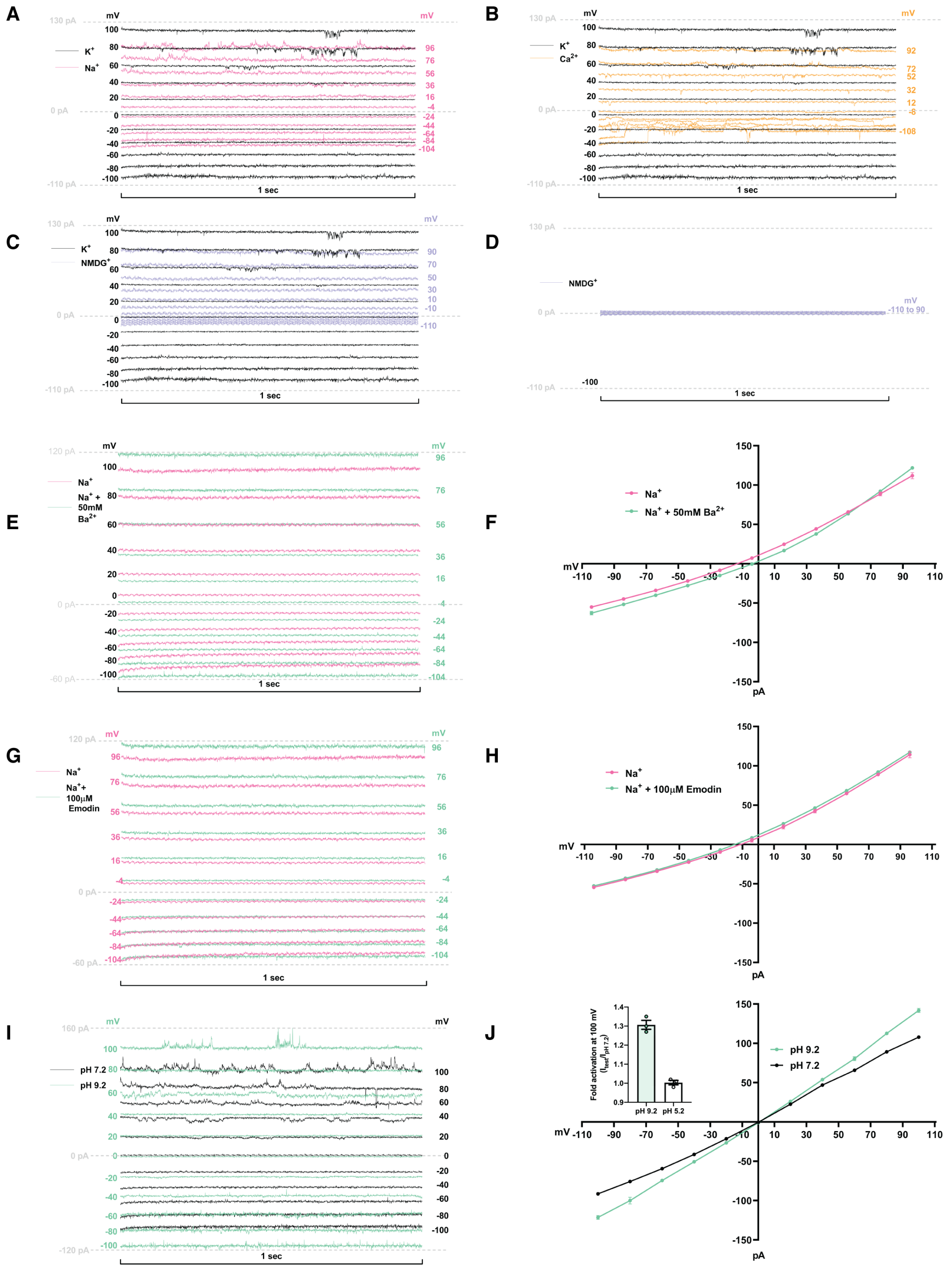

Figure S2 - Patch recordings from 3a-proteoliposomes (A-E, G, I) Current recordings from 3a-proteoliposome patches with 150 mM K^+ pipette solution. Each panel is from a single patch. (A) 150 mM K^+ (black) or 150 mM Na^+ (pink) bath solution. (B) 150 mM K^+ (black) or 75 mM Ca^{2+} (orange) bath solution. (C) K^+ (black) or 150 mM $NMDG^+$ (blue) bath solution. (D) 150 mM $NMDG^+$ (blue) bath solution, but after all channels had closed or inactivated. (E) 150 mM Na^+ (pink) or 150 mM Na^+ with 50 mM Ba^{2+} (green) bath solution. (F) Current-voltage relationship plotted from (E). (G) 150 mM Na^+ (pink) or 150 mM Na^+ with 100 µM emodin (green) bath solution. (H) Current-voltage relationship plotted from (G). (I) 150 mM K^+ pH 7.2 (black) or 150 mM K^+ pH 9.2 (green) bath solution. (J) Current-voltage relationship plotted from (I). (J, inset) Fold activation at +100 mV at pH 9.2 or pH 5.2 compared to pH 7.2. Currents were recorded with the following protocol: $V_{hold} = 0$ mV, $V_{test} = -100$ to 100 mV, $\Delta V = 20$ mV, $t_{test}=1$ sec. Voltages indicated were corrected after recording for liquid junction potential.

bioRxiv preprint doi: https://doi.org/10.1101/2020.06.17.156554. this version posted June 25, 2020. The copyright holder for this preprint (which was not certified by peer review) is the author/funder. It is made available under a CC-BY 4.0 International license.

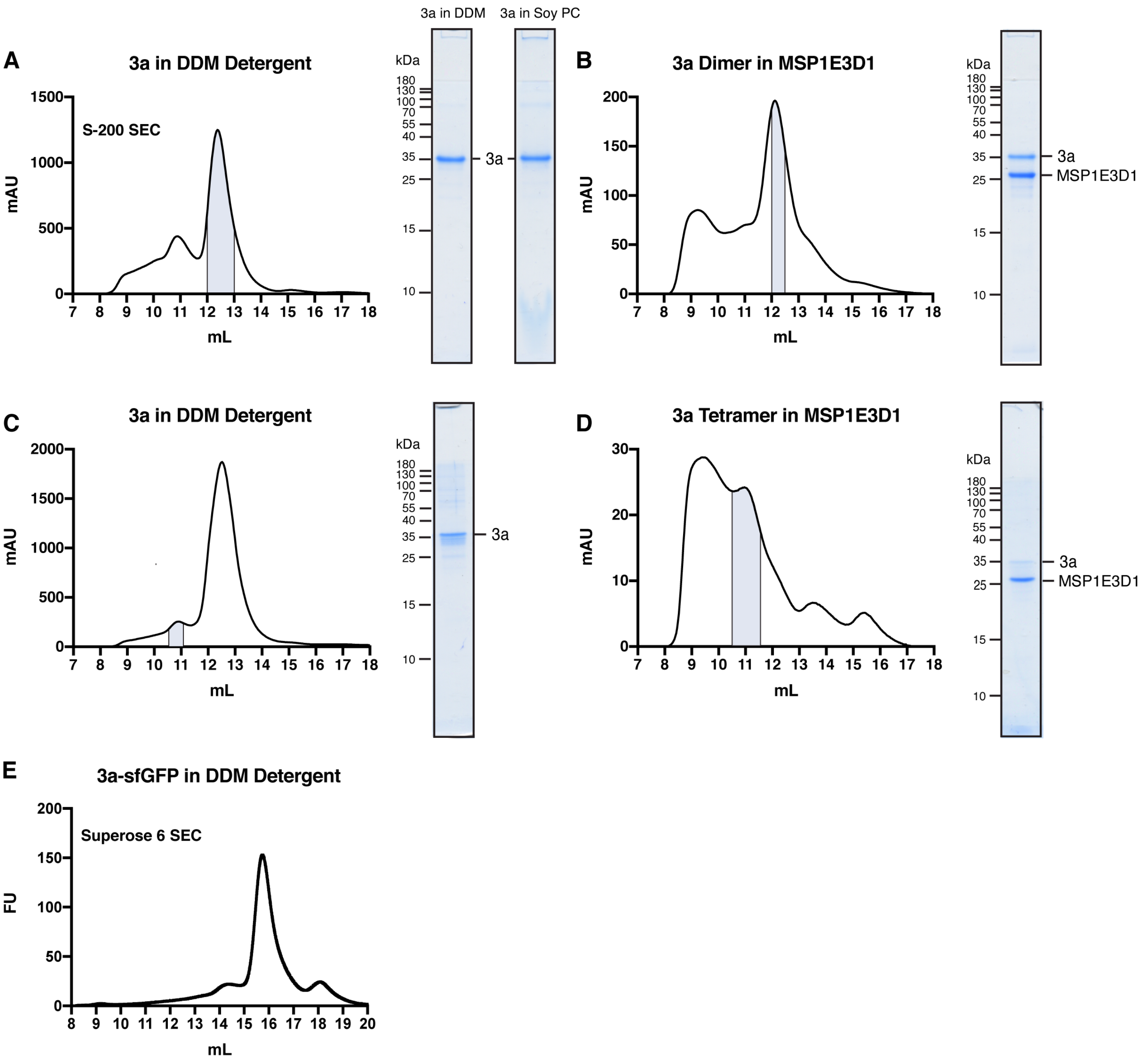

Figure S3 – Purification and reconstitution of 3a (A) Size exclusion chromatogram of 3a expressed in insect cells and extracted and purified in DDM (left). Pooled fractions corresponding to dimeric 3a are highlighted in blue. Coomassie-stained SDS-PAGE of pooled dimeric 3a-containing fractions (center) and of 3a following reconstitution into PC lipids (right). (B) Size exclusion chromatogram of dimeric 3a reconstituted into MSP1E3D1 lipid nanodiscs (left). Pooled fractions are highlighted blue. (C,D) Same as (A,B), but for tetrameric 3a. (E) GFP fluorescence chromatogram of 3a expressed in SF9 cells and extracted in DDM detergent. Samples were run on a Superose 6 column.

bioRxiv preprint doi: https://doi.org/10.1101/2020.06.17.156554. this version posted June 25, 2020. The copyright holder for this preprint (which was not certified by peer review) is the author/funder. It is made available under a CC-BY 4.0 International license.

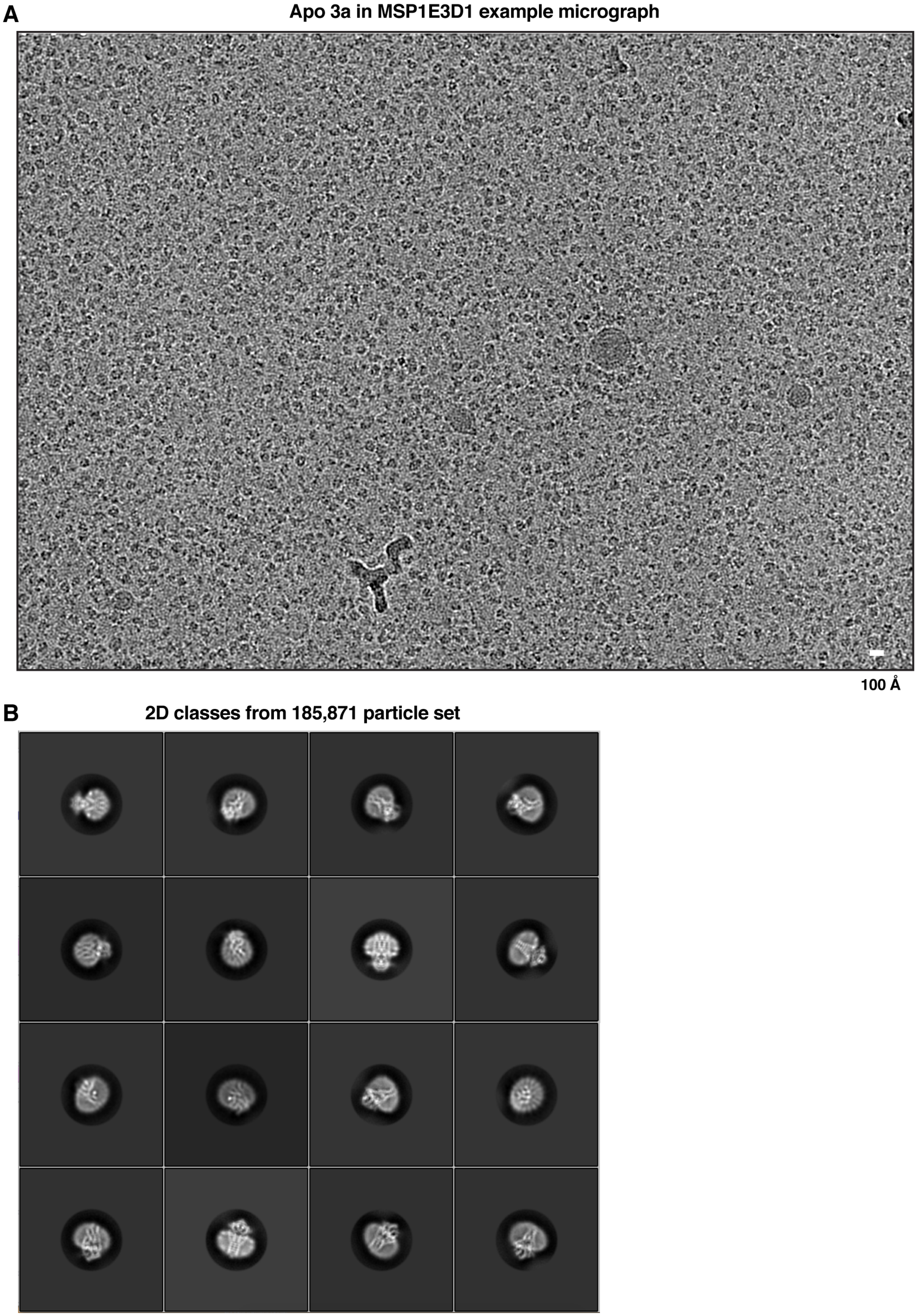

Figure S4 - Example micrographs and 2D class averages of dimeric apo 3a in MSP1E3D1 lipid nanodiscs (A) Representative micrograph and (B) 2D class averages of dimeric apo 3a in MSP1E3D1 lipid nanodiscs.

bioRxiv preprint doi: https://doi.org/10.1101/2020.06.17.156554. this version posted June 25, 2020. The copyright holder for this preprint (which was not certified by peer review) is the author/funder. It is made available under a CC-BY 4.0 International license.

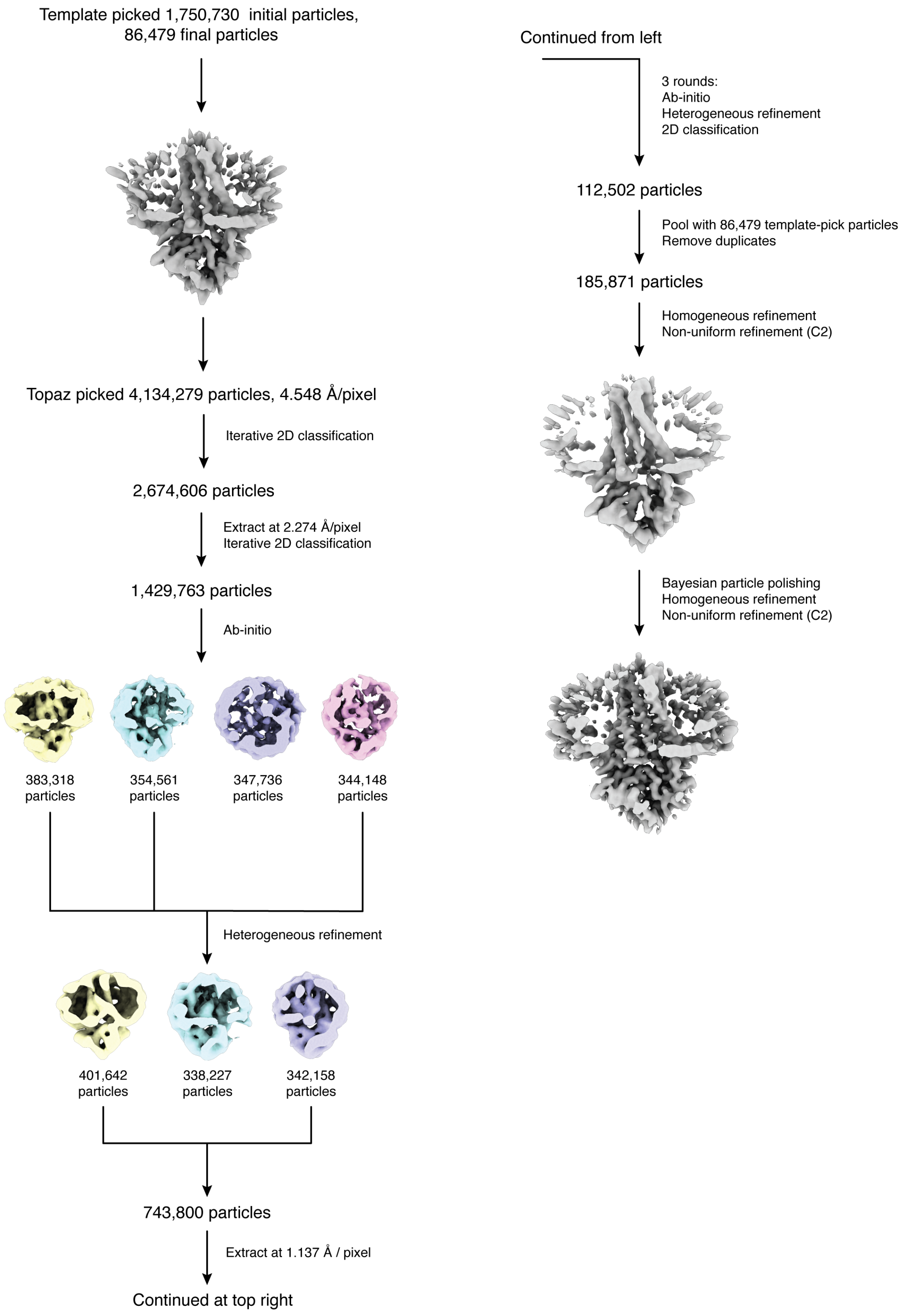

Figure S5 - Cryo-EM processing pipeline for dimeric apo 3a in MSP1E3D1 lipid nanodiscs Overview of Cryo-EM data processing pipeline in cryoSPARC and Relion. See Methods for details.

bioRxiv preprint doi: https://doi.org/10.1101/2020.06.17.156554. this version posted June 25, 2020. The copyright holder for this preprint (which was not certified by peer review) is the author/funder. It is made available under a CC-BY 4.0 International license.

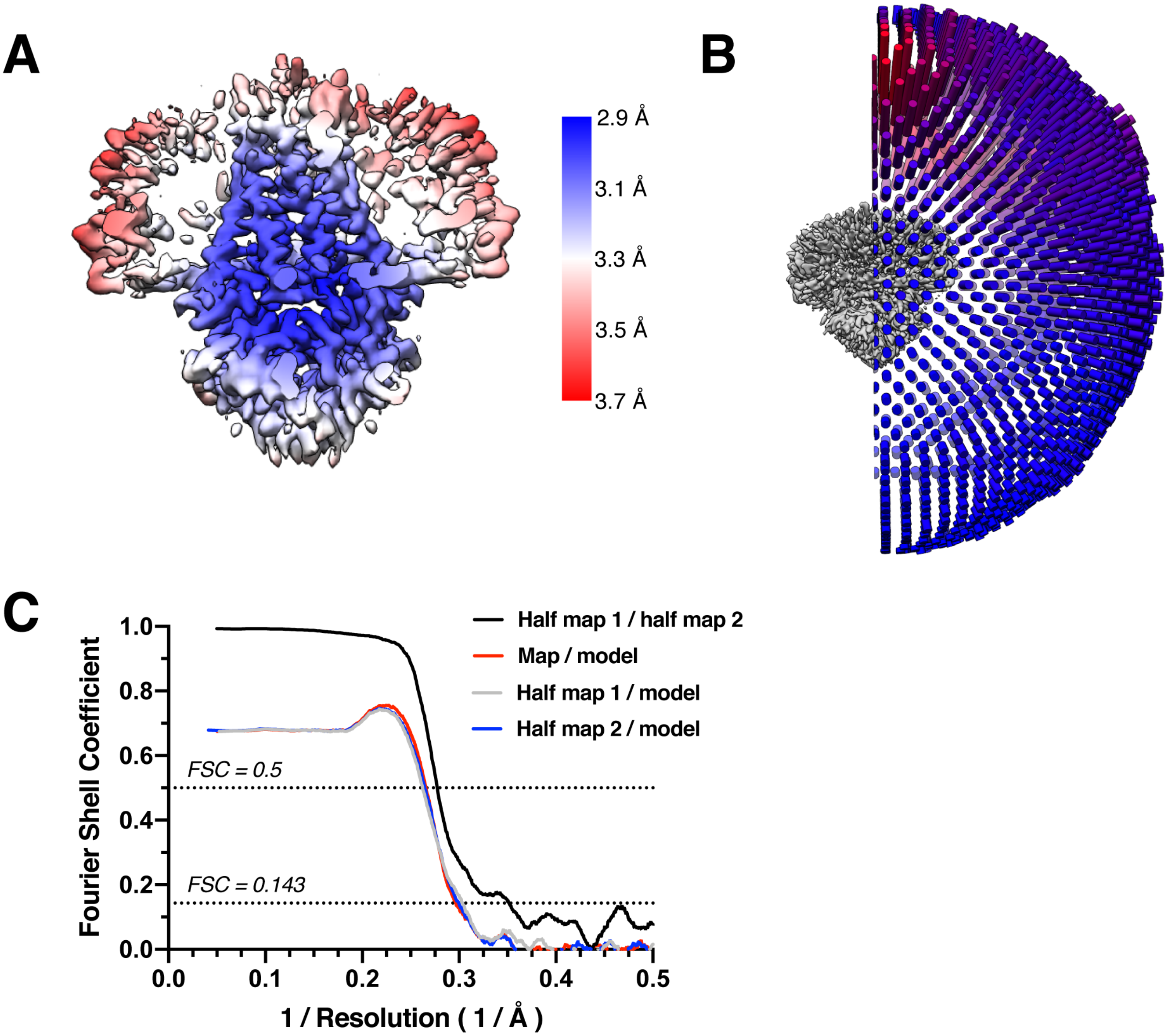

Figure S6 - Cryo-EM validation for dimeric apo 3a in MSP1E3D1 lipid nanodiscs (A) Local resolution estimated in Relion colored as indicated on the final map. (B) Angular distribution of particles used in final refinement with final map for reference. (C) Fourier Shell Correlation (FSC) relationships between (black) the two unfiltered half-maps from refinement and used for calculating overall resolution at 0.143, (red) the final map and model, (gray) half-map one and model, and (blue) half-map and model.

bioRxiv preprint doi: https://doi.org/10.1101/2020.06.17.156554. this version posted June 25, 2020. The copyright holder for this preprint (which was not certified by peer review) is the author/funder. It is made available under a CC-BY 4.0 International license.

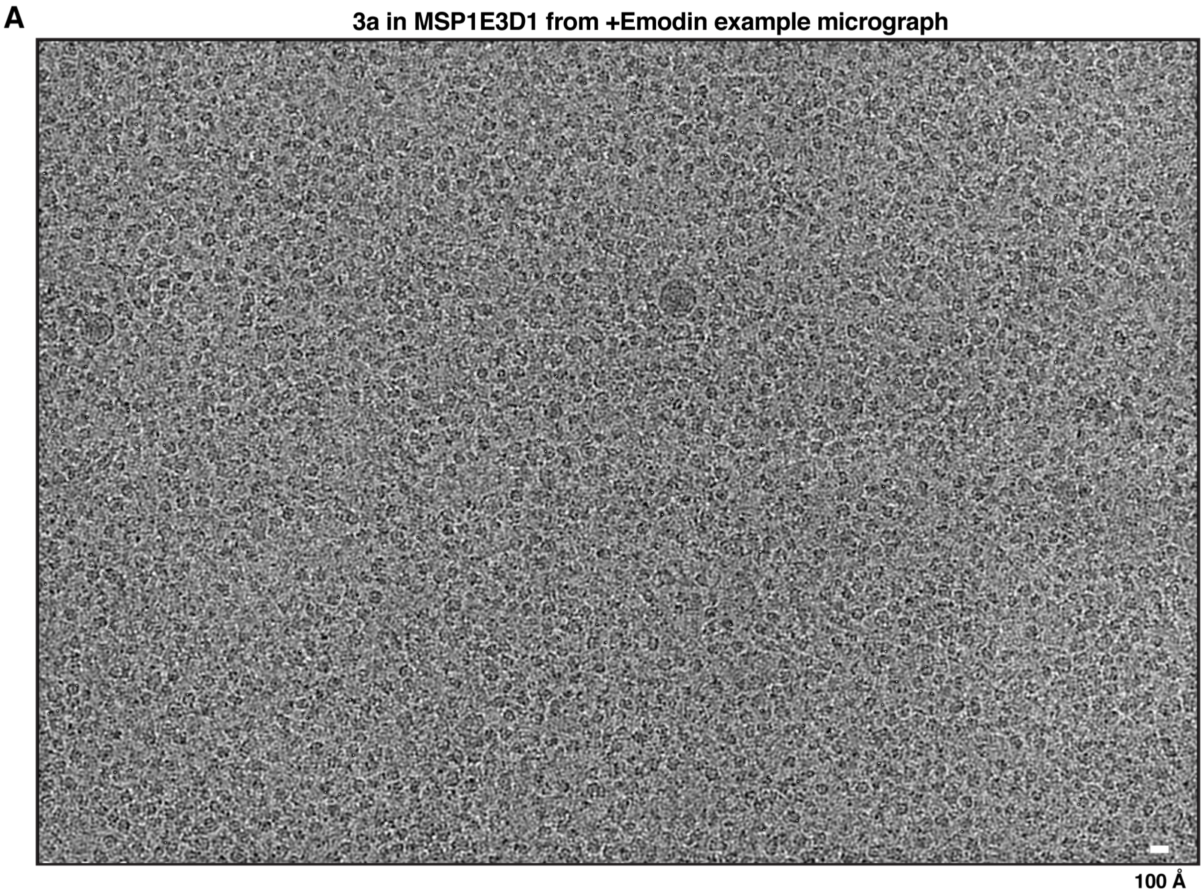

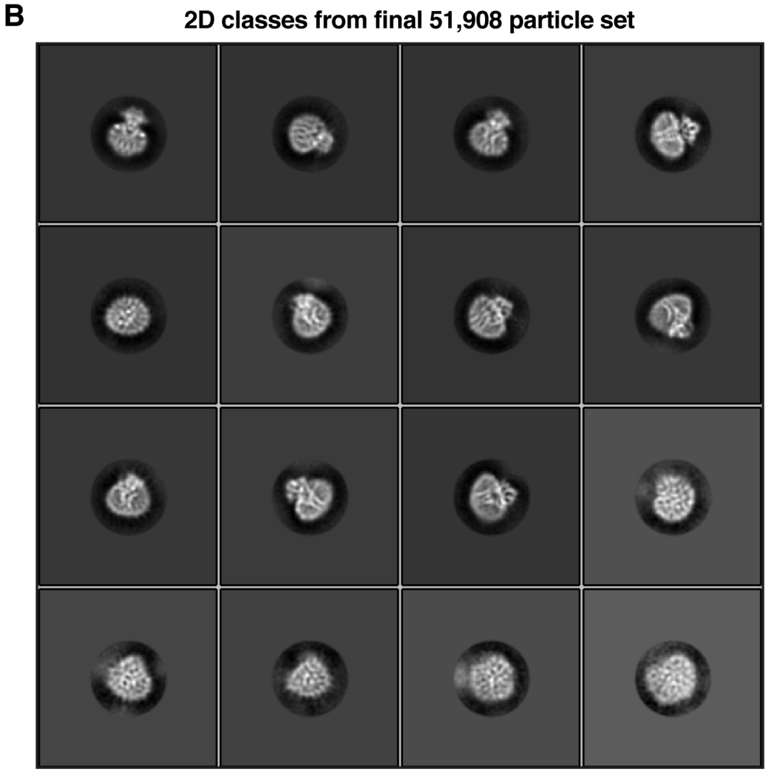

Figure S7 - Example micrographs and 2D class averages of dimeric 3a in MSP1E3D1 lipid nanodiscs with emodin added (A) Representative micrograph and (B) 2D class averages of dimeric 3a in MSP1E3D1 lipid nanodiscs with emodin added.

bioRxiv preprint doi: https://doi.org/10.1101/2020.06.17.156554. this version posted June 25, 2020. The copyright holder for this preprint (which was not certified by peer review) is the author/funder. It is made available under a CC-BY 4.0 International license.

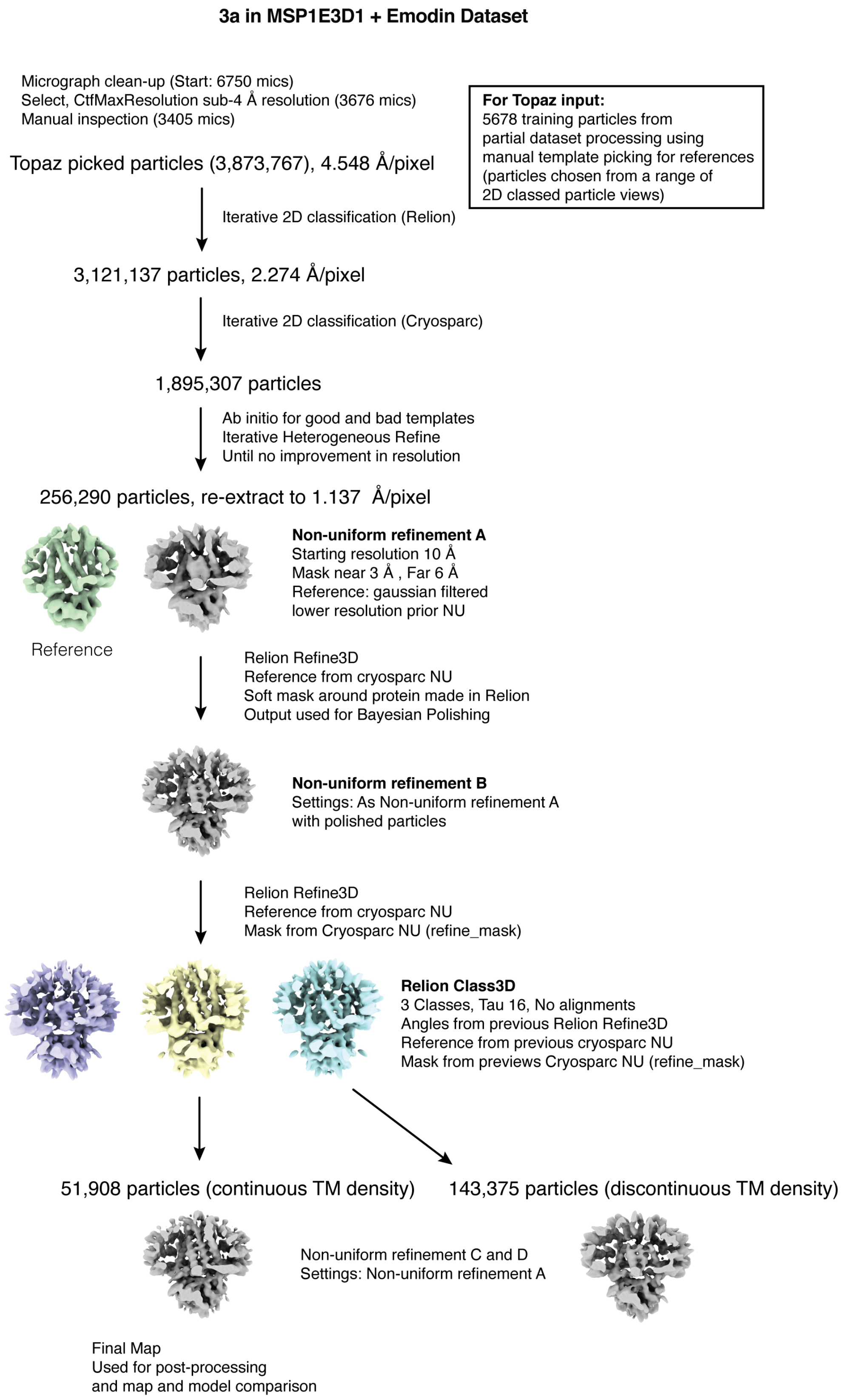

Figure S8 - Cryo-EM processing pipeline for dimeric 3a in MSP1E3D1 lipid nanodiscs with emodin added Overview of Cryo-EM data processing pipeline in cryoSPARC and Relion. See Methods for details.

bioRxiv preprint doi: https://doi.org/10.1101/2020.06.17.156554. this version posted June 25, 2020. The copyright holder for this preprint (which was not certified by peer review) is the author/funder. It is made available under a CC-BY 4.0 International license.

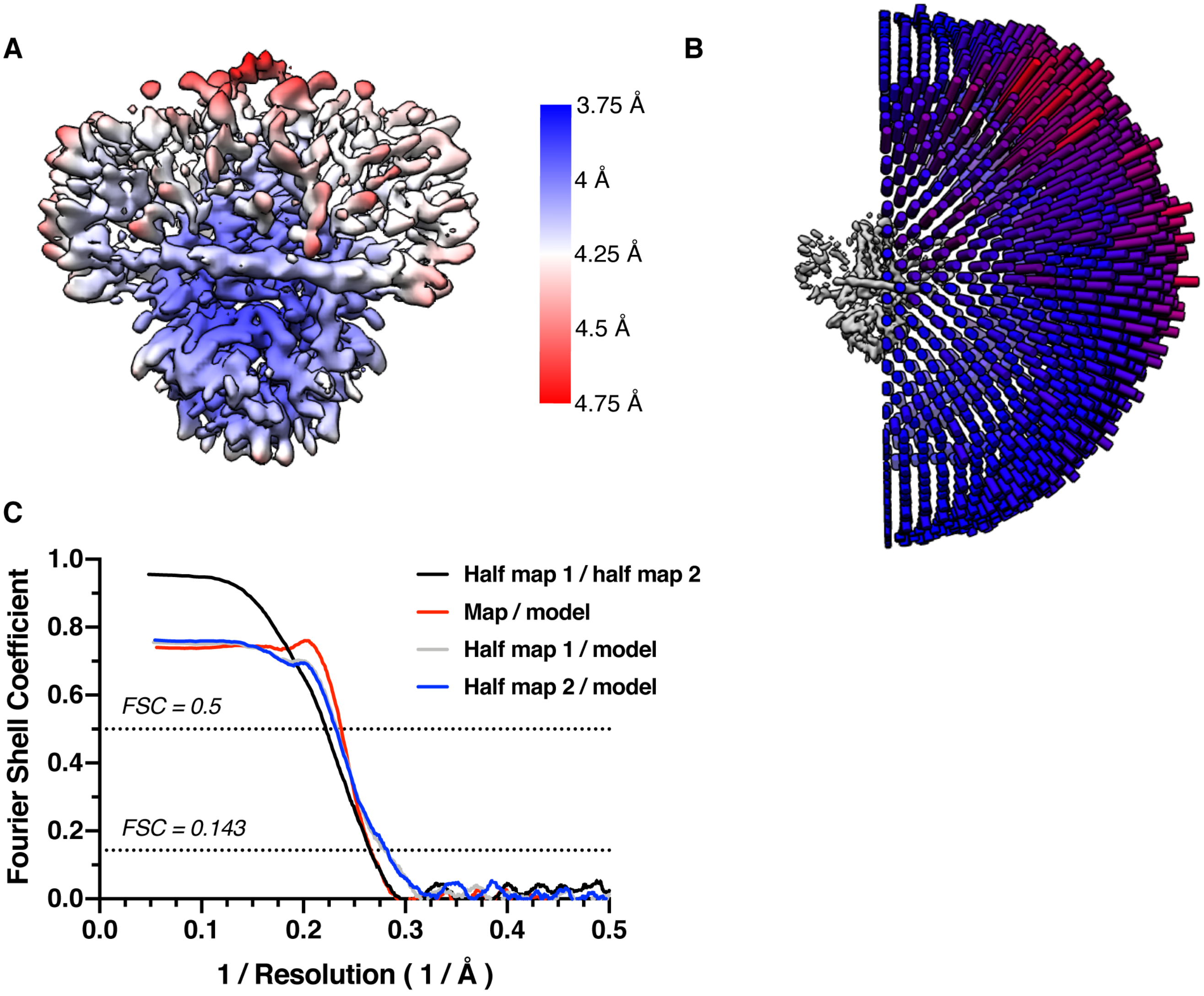

Figure S9 - Cryo-EM validation for dimeric 3a in MSP1E3D1 lipid nanodiscs with emodin added (A) Local resolution estimated in Relion colored as indicated on the final map. (B) Angular distribution of particles used in final refinement with final map for reference. (C) Fourier Shell Correlation (FSC) relationships between (black) the two unfiltered half-maps from refinement and used for calculating overall resolution at 0.143, (red) the final map and model, (gray) half-map one and model, and (blue) half-map and model.

bioRxiv preprint doi: https://doi.org/10.1101/2020.06.17.156554. this version posted June 25, 2020. The copyright holder for this preprint (which was not certified by peer review) is the author/funder. It is made available under a CC-BY 4.0 International license.

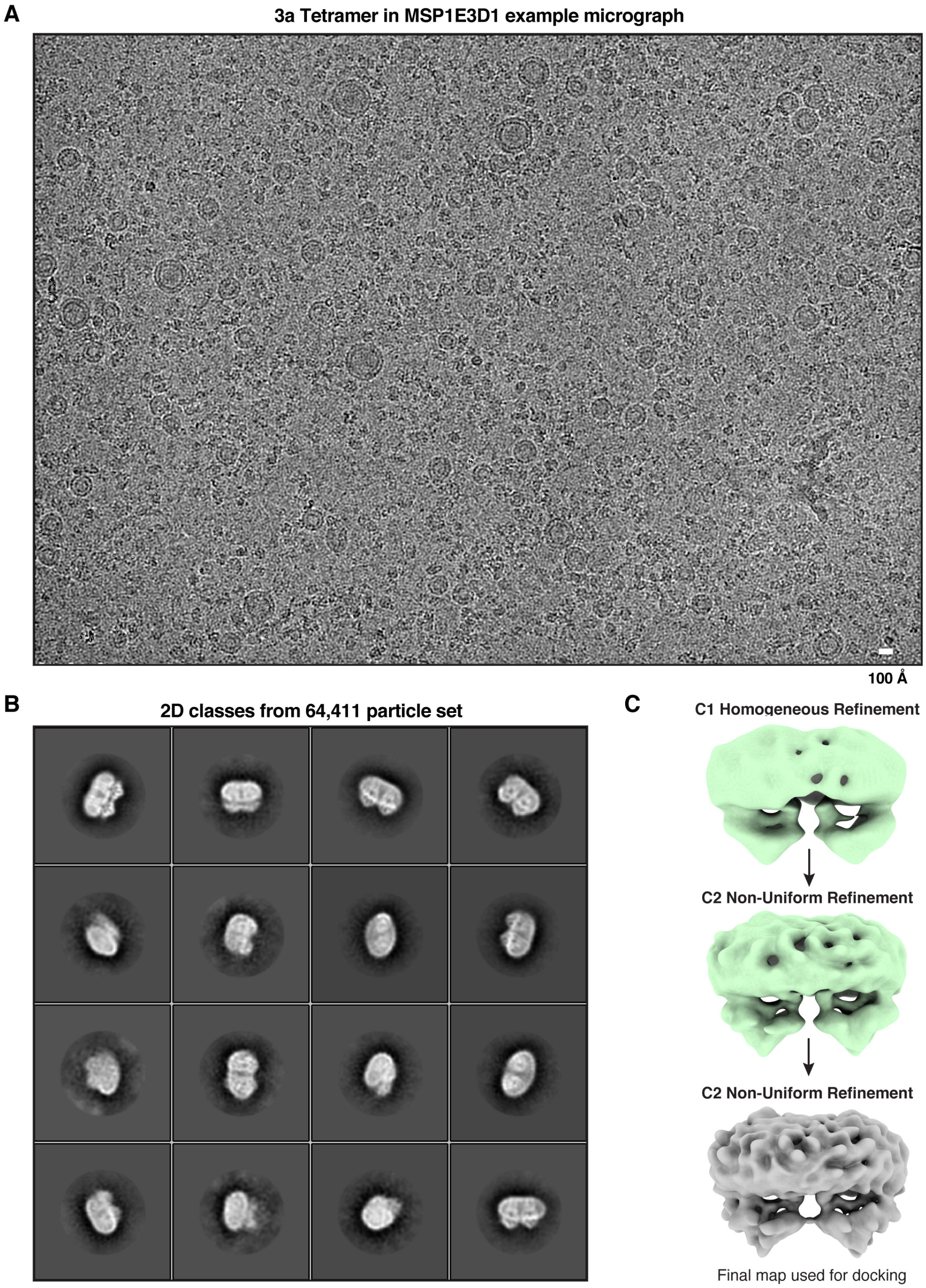

Figure S10 - Example micrographs and 2D class averages of tetrameric apo 3a in MSP1E3D1 lipid nanodiscs (A) Representative micrograph and (B) 2D class averages of tetrameric apo 3a in MSP1E3D1 lipid nanodiscs. (C) Map overview pipeline for final steps of processing (Also see Methods).

bioRxiv preprint doi: https://doi.org/10.1101/2020.06.17.156554. this version posted June 25, 2020. The copyright holder for this preprint (which was not certified by peer review) is the author/funder. It is made available under a CC-BY 4.0 International license.

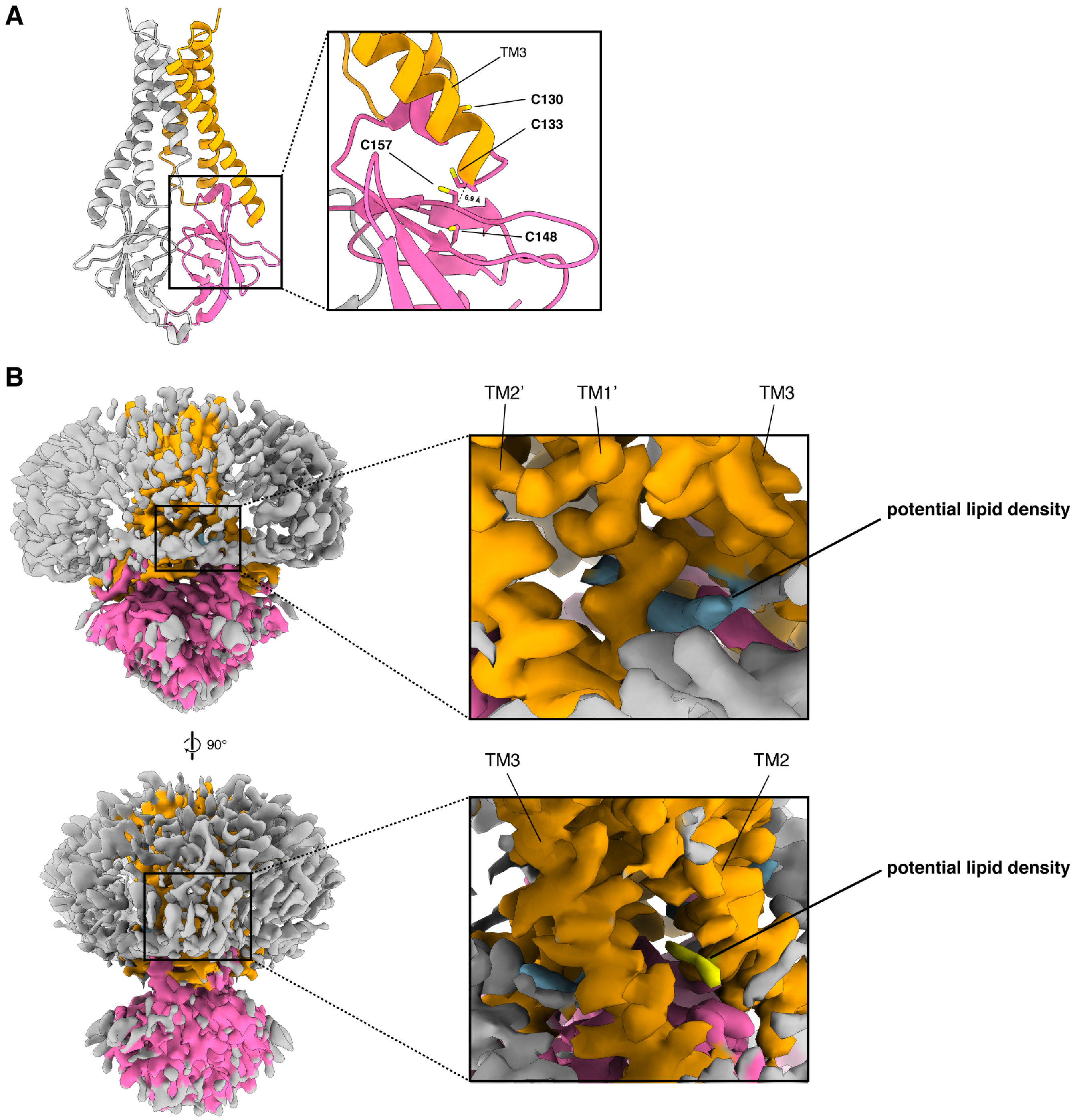

Figure S11 – Structural elements in dimeric 3a (A) Full model (left) with boxed region zoom-in (right) to show the cysteine-rich region of 3a. (B) Tubular unassigned lipid-like densities in the apo 3a map, colored blue and yellow. Displayed at threshold 0.7, with surface dust hidden (size 6).

bioRxiv preprint doi: https://doi.org/10.1101/2020.06.17.156554. this version posted June 25, 2020. The copyright holder for this preprint (which was not certified by peer review) is the author/funder. It is made available under a CC-BY 4.0 International license.

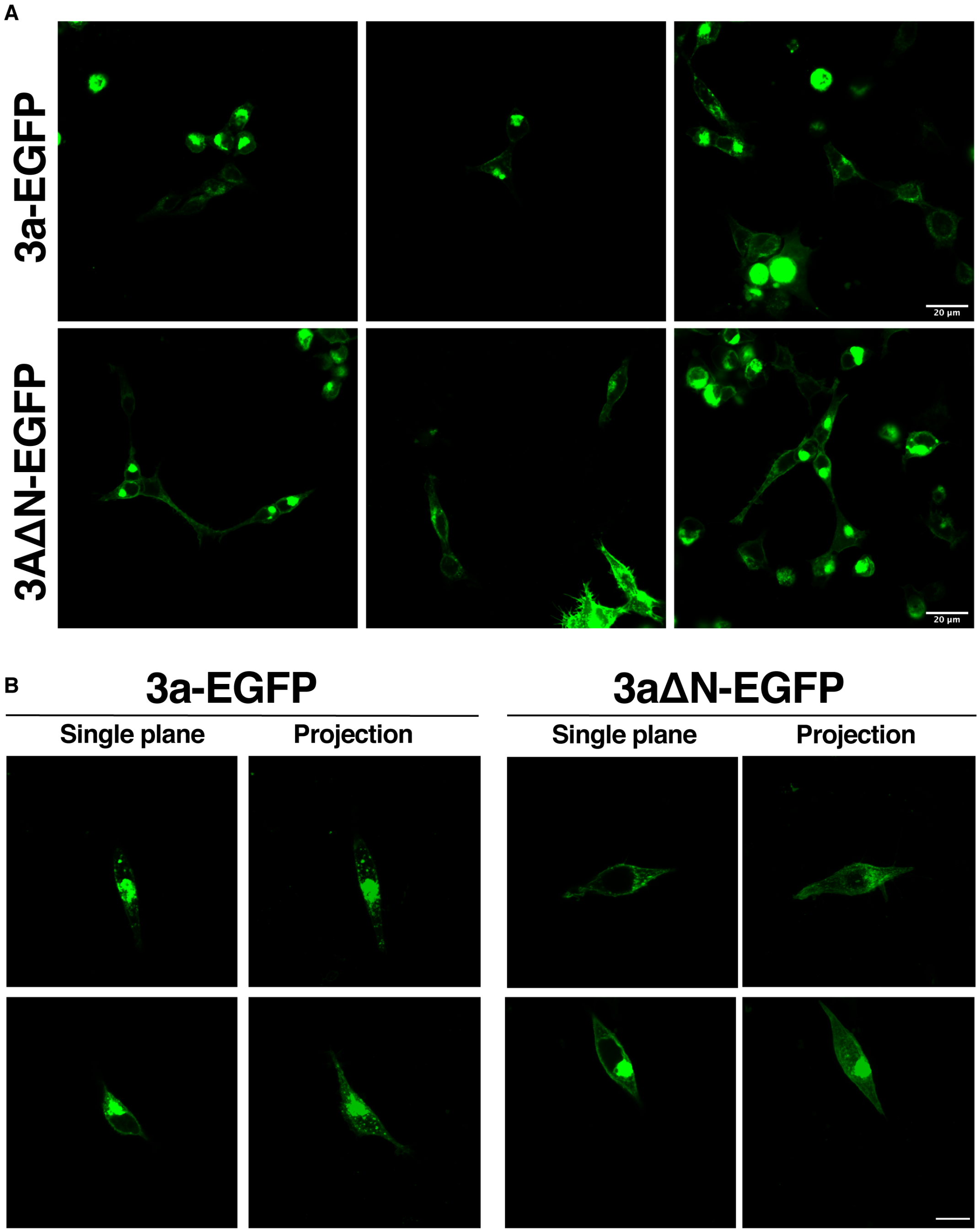

Figure S12 – Localization of 3a-EGFP and 3aΔN-EGFP expressed in HEK cells (A) Field of view with multiple cells imaged with 20X objective. Scale bar, 20 μm. (B) Cells imaged with 63X objective with both single plane and brightest-point projections displayed. Scale bar, 10 μm.

bioRxiv preprint doi: https://doi.org/10.1101/2020.06.17.156554. this version posted June 25, 2020. The copyright holder for this preprint (which was not certified by peer review) is the author/funder. It is made available under a CC-BY 4.0 International license.

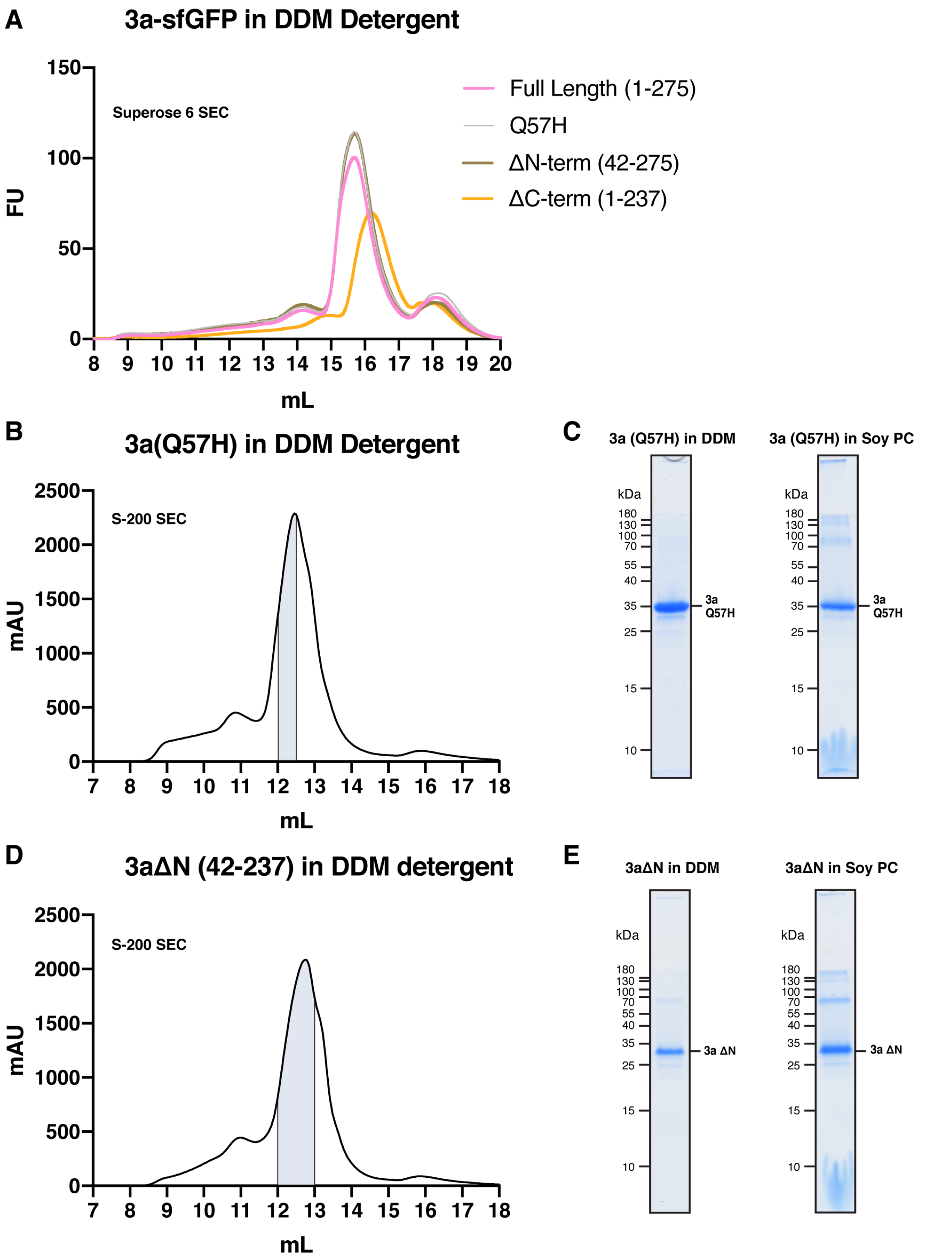

Figure S13 – Purification and reconstitution of 3aΔN and 3a Q57H (A) GFP fluorescence chromatogram of 3a, as well as mutants and truncations, expressed in SF9 cells and extracted in DDM detergent. Samples were run on a Superose 6 column. (B) Size exclusion chromatogram of 3a Q57H expressed in insect cells and extracted and purified in DDM (left). Pooled fractions corresponding to dimeric 3a are highlighted in blue. (C) Coomassie-stained SDS-PAGE of pooled dimeric 3a Q57H -containing fractions (center) and of 3a following reconstitution into PC lipids (right). (D,E) Same as (B,C) but for 3aΔN.

bioRxiv preprint doi: https://doi.org/10.1101/2020.06.17.156554. this version posted June 25, 2020. The copyright holder for this preprint (which was not certified by peer review) is the author/funder. It is made available under a CC-BY 4.0 International license.

A

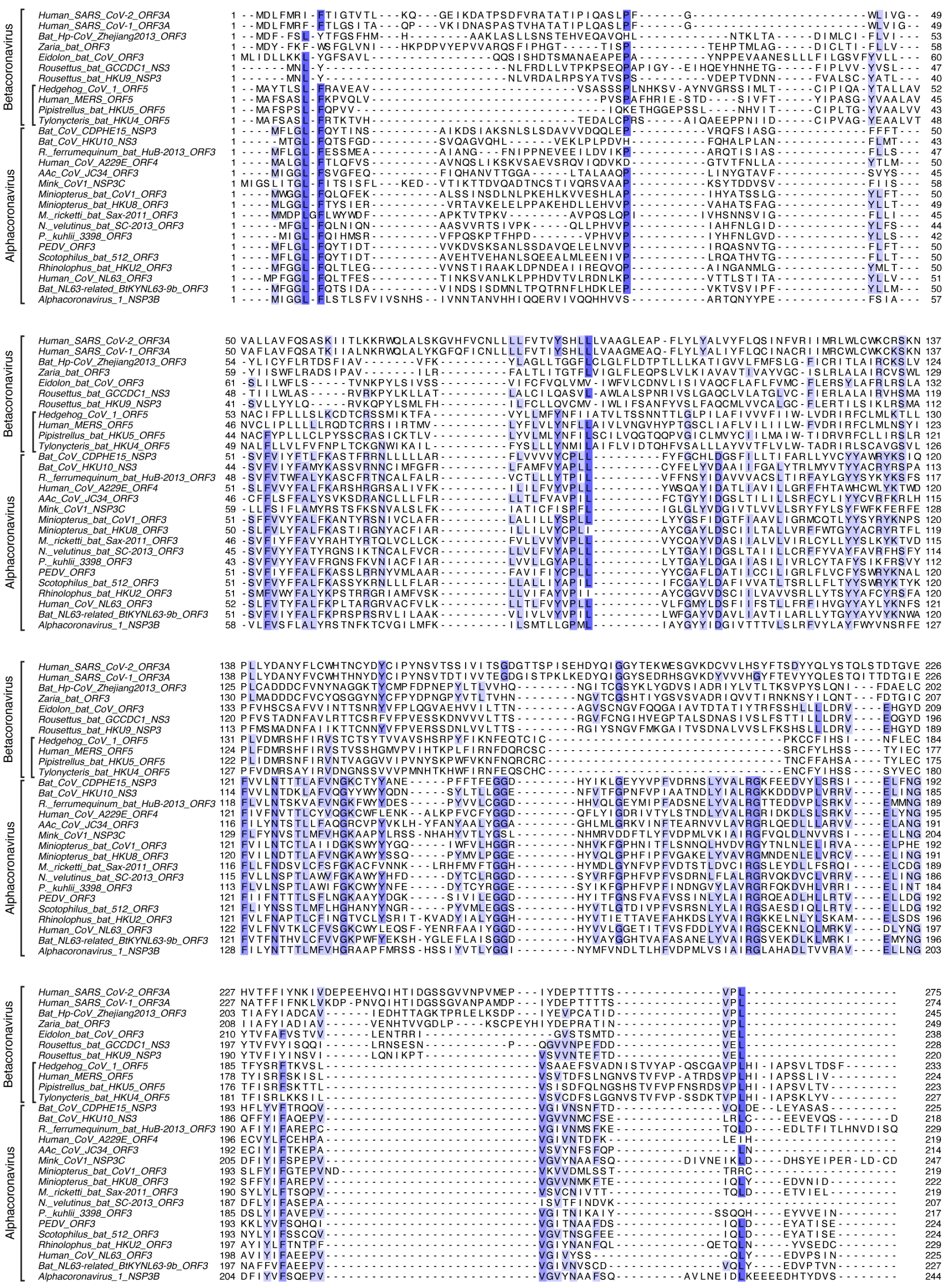

B

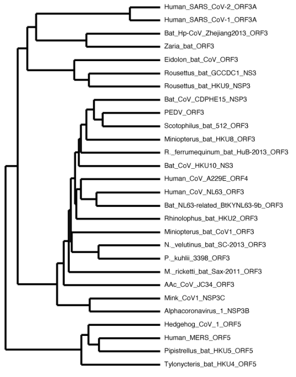

Figure S14 - Sequence alignment of 3a-like proteins across *Coronaviridae* Legend continued on following page.

bioRxiv preprint doi: https://doi.org/10.1101/2020.06.17.156554. this version posted June 25, 2020. The copyright holder for this preprint (which was not certified by peer review) is the author/funder. It is made available under a CC-BY 4.0 International license.

Figure S14 - Sequence alignment of 3a-like proteins across *Coronaviridae* (A) Alignment of twenty-eight 3a-like protein sequences colored by conservation in a ramp from white (not conserved) to dark blue (highly conserved). Accession numbers are listed in Table 2. Sequences were selected from representative species from each Coronavirus subgenus. *Alphacoronavirus* and *Betacoronavirus* clades are indicated. Within B*etacoronavirus* the subgenus *Merbecovirus* is also indicated with a bar. (B) Neighbor-joining tree calculated from the alignment in (A).
